# Novel dTDP-l-Rhamnose Synthetic Enzymes (RmlABCD) From *Saccharothrix syringae* CGMCC 4.1716 for One-Pot Four-Enzyme Synthesis of dTDP-l-Rhamnose

**DOI:** 10.3389/fmicb.2021.772839

**Published:** 2021-11-08

**Authors:** Shida Yang, Xiaonan An, Guofeng Gu, Zhenxin Yan, Xukai Jiang, Li Xu, Min Xiao

**Affiliations:** ^1^State Key Laboratory of Microbial Technology, Shandong University, Qingdao, China; ^2^National Glycoengineering Research Center, Shandong Key Laboratory of Carbohydrate Chemistry and Glycobiology, Shandong University, Qingdao, China; ^3^NMPA Key Laboratory for Quality Research and Evaluation of Carbohydrate-based Medicine Shandong University, Qingdao, China

**Keywords:** RmlABCD enzymes, *Saccharothrix syringae*, dTDP-l-rhamnose, dUDP-l-rhamnose, enzymatic synthesis

## Abstract

Deoxythymidine diphospho-l-rhamnose (dTDP-l-rhamnose) is used by prokaryotic rhamnosyltransferases as the glycosyl donor for the synthesis of rhamnose-containing polysaccharides and compounds that have potential in pharmaceutical development, so its efficient synthesis has attracted much attention. In this study, we successfully cloned four putative dTDP-l-rhamnose synthesis genes *Ss-rmlABCD* from *Saccharothrix syringae* CGMCC 4.1716 and expressed them in *Escherichia coli*. The recombinant enzymes, Ss-RmlA (glucose-1-phosphate thymidylyltransferase), Ss-RmlB (dTDP-d-glucose 4,6-dehydratase), Ss-RmlC (dTDP-4-keto-6-deoxy-glucose 3,5-epimerase), and Ss-RmlD (dTDP-4-keto-rhamnose reductase), were confirmed to catalyze the sequential formation of dTDP-l-rhamnose from deoxythymidine triphosphate (dTTP) and glucose-1-phosphate (Glc-1-P). Ss-RmlA showed maximal enzyme activity at 37°C and pH 9.0 with 2.5mMMg^2+^, and the *K*_m_ and *k*_cat_ values for dTTP and Glc-1-P were 49.56μM and 5.39s^−1^, and 117.30μM and 3.46s^−1^, respectively. Ss-RmlA was promiscuous in the substrate choice and it could use three nucleoside triphosphates (dTTP, dUTP, and UTP) and three sugar-1-Ps (Glc-1-P, GlcNH_2_-1-P, and GlcN_3_-1-P) to form nine sugar nucleotides (dTDP-GlcNH_2_, dTDP-GlcN_3_, UDP-Glc, UDP-GlcNH_2_, UDP-GlcN_3_, dUDP-Glc, dUDP-GlcNH_2_, and dUDP-GlcN_3_). Ss-RmlB showed maximal enzyme activity at 50°C and pH 7.5 with 0.02mM NAD^+^, and the *K*_m_ and *k*_cat_ values for dTDP-glucose were 98.60μM and 11.2s^−1^, respectively. A one-pot four-enzyme reaction system was developed by simultaneously mixing all of the substrates, reagents, and four enzymes Ss-RmlABCD in one pot for the synthesis of dTDP-l-rhamnose and dUDP-l-rhamnose with the maximal yield of 65% and 46%, respectively, under the optimal conditions. dUDP-l-rhamnose was a novel nucleotide-activated rhamnose reported for the first time.

## Introduction

L-rhamnose is a common 6-deoxy hexose found in the cell wall polysaccharides of many bacteria and the carbohydrate moieties of many natural products from bacteria and plants (Halimah et al., 2015; Khalil et al., 2015; Wittgens et al., 2018; Edgar et al., 2019; Garcia-Vello et al., 2020). These rhamnose-containing biomolecules show great potential in the development of drugs and vaccines owing to their distinct bioactive properties (Wang et al., 2011; Lee et al., 2014; Mei et al., 2019; Pequegnat and Monteiro, 2019). Moreover, the anti-rhamnose antibody present in high titer in human serum can specifically bind to rhamnose or rhamnose-containing compounds, which inspires the design of antitumor vaccines conjugated with rhamnose for augmenting immunogenicity based on an antibody-dependent antigen uptake mechanism (Sarkar et al., 2010; Hossain et al., 2018). Therefore, the synthesis of rhamnose-containing biomolecules with pharmacological effects has been considered to be of great importance and attracted much attention (Xing et al., 2015; Sylla et al., 2019; Xu et al., 2019).

**Figure 1:**
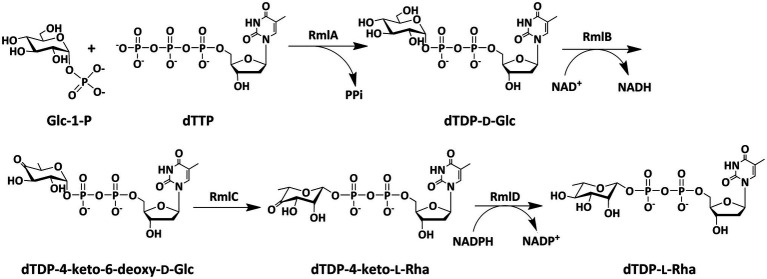
Biosynthetic pathway of dTDP-l-rhamnose in bacteria and archaea.

Since bacterial rhamnosyltransferases are usually promiscuous in their substrate choice and highly efficient in catalysis, they are recognized as desirable biocatalysts in the synthesis of rhamnose-containing biomolecules (Cha et al., 2008; Chen et al., 2009; Strobel et al., 2013; Nguyen et al., 2020). However, dTDP-l-rhamnose, a key substrate as the rhamnosyl donor for these enzymes, is difficult to obtain from either natural resource extraction or chemical synthesis (Li et al., 2016; Rai and Kulkarni, 2020). In nature, most of sugar nucleotides are biosynthesized in a one-step reaction by the catalysis of appropriate sugar nucleotidylyltransferases from nucleoside triphosphates (NTPs) and sugar-1-phosphates (sugar-1-Ps; Guan et al., 2009; Li et al., 2011, 2021), whereas the biosynthesis of dTDP-l-rhamnose requires four steps of reactions, including thymidylyl transfer, dehydration, epimerization, and reduction, which poses a challenge to the efficient preparation of this sugar nucleotide (Kaysser et al., 2010; Dhaked et al., 2019; Li et al., 2021). In bacteria and archaea, dTDP-l-rhamnose is consecutively synthesized by four enzymes, RmlA, RmlB, RmlC, and RmlD, starting from Glc-1-P and dTTP (Figure 1). RmlA (E.C. 2.7.7.24) is a Glc-1-P thymidylyltransferase that catalyzes the formation of dTDP-d-glucose (dTDP-d-Glc) from Glc-1-P and dTTP. RmlB (E.C.4.2.1.46) is a dTDP-d-glucose 4,6-dehydratase that dehydrates dTDP-d-glucose in the presence of NAD^+^ to form dTDP-4-keto-6-deoxy-glucose. RmlC (E.C.5.1.3.13) is a dTDP-4-keto-6-deoxy-glucose 3,5-epimerase that catalyzes the double epimerization of dTDP-4-keto-6-deoxy-glucose to form dTDP-4-keto-rhamnose. RmlD (E.C.1.1.1.133) is a dTDP-4-keto-rhamnose reductase that reduces dTDP-4-keto-rhamnose to dTDP-l-rhamnose in the presence of NADPH.

Over the past few decades, many Rml enzymes from different prokaryotes have been reported (Graninger et al., 2002; Han et al., 2007; Steiner et al., 2008; van der Beek et al., 2019). Most of those works focused on the biological roles of these enzymes and their potential as drug targets for the treatment of pathogenic bacteria, such as *Mycobacteria tuberculosis* H37Rv (Brown et al., 2017), *Streptococcus pyogenes* 5,448 (van der Beek et al., 2019), *Bacillus anthracis* str. Ames (Gokey et al., 2018), etc. However, there are a few studies on the *in vitro* synthesis of dTDP-l-rhamnose using these enzymes.

Several efforts have been made in the enzymatic synthesis of dTDP-l-rhamnose using Rml enzymes from different bacteria. A two-step synthesis of dTDP-l-rhamnose from dTDP-d-glucose was achieved using RmlBCD from *Salmonella enterica* LT2 (Marumo et al., 1992). This method first used RmlB to catalyze the conversion of dTDP-d-glucose into dTDP-4-keto-6-deoxy-glucose, and then RmlC and RmlD were added to further catalyze the formation of dTDP-l-rhamnose. Another one-pot synthesis of dTDP-l-rhamnose from dTDP-d-glucose was accomplished with RmlBCD from *Aneurinibacillus thermoaerophilus* DSM 10155 (Pazur and Shuey, 2002). These two approaches employed the expensive substrate dTDP-d-glucose, which limited the scale of production of dTDP-l-rhamnose. A low-cost synthesis of dTDP-l-rhamnose from dTMP and Glc-1-P was achieved by a combined enzymatic pathway with six crude enzymes, in which three enzymes of TMP kinase, acetate kinase, and dTDP-glucose synthase (RmlA) were cloned from *Escherichia coli*, RmlB was cloned from *Salmonella ente*rica LT2, and two enzymes of RmlC and RmlD were from *Mesorhizobium loti* (Oh et al., 2003; Kang et al., 2006). However, when using cell extracts as catalysts, the replicability of the method and the purification of product were challenging. Another two-step synthesis of dTDP-l-rhamnose was conducted using five enzymes from dTMP and sucrose (Elling et al., 2005). This method first used dTMP-kinase from *Saccharomyces cerevisiae*, sucrose synthase from potato, and RmlB from *S. ente*rica LT2 to catalyze the synthesis of dTDP-4-keto-6-deoxy-glucose from dTMP and sucrose, and then the purified dTDP-4-keto-6-deoxy-glucose was used as substrate in the reaction catalyzed by RmlC and RmlD from *S. ente*rica LT2 to generate dTDP-l-rhamnose. This method started synthesis from cheap substrates but the operation was complicated. Recently, a one-pot four-enzyme synthesis of dTDP-l-rhamnose with Cps23FL, Cps23FM, Cps23FN, and Cps23FO from *Streptococcus pneumoniae* 23F was developed by the addition of the enzymes in two portions, in which, in order to avoid the probable inhibitory effect of dTDP-l-rhamnose on the enzyme activity, Cps23FL was mixed with the substrates and reagents for the formation of dTDP-d-glucose firstly, and then the other three enzymes, Cps23FM, Cps23FN, and Cps23FO, were added for further catalytic reaction to produce dTDP-l-rhamnose (Li et al., 2016). From all the published work mentioned above, it could be seen that the discovery of new Rml enzymes and the development of a simple and efficient reaction system for the practical enzymatic synthesis of dTDP-l-rhamnose should be of great significance.

In this work, four putative genes *Ss-rmlABCD* encoding for dTDP-l-rhamnose biosynthesis pathway in *Saccharothrix syringae* CGMCC 4.1716 were successfully cloned and expressed in *E. coli* BL21 (DE3), and the sequential synthesis of dTDP-l-rhamnose by these four enzymes Ss-RmlABCD was proved. The enzymatic properties of Ss-RmlA and Ss-RmlB, the first two enzymes in the dTDP-l-rhamnose biosynthesis pathway, were studied in detail. A new simple and efficient four-enzyme reaction system for the synthesis of dTDP-l-rhamnose by simultaneously mixing all of the substrates, reagents, and four enzymes Ss-RmlABCD in one pot was then developed and optimized. This reaction system could also convert Glc-1-P and dUTP to dUDP-l-rhamnose. Our work characterized a novel set of dTDP-l-rhamnose synthesis enzymes derived from *S. syringae* CGMCC 4.1716 and further revealed its potential as the biocatalyst for the practical enzymatic synthesis of dTDP-l-rhamnose and dUDP-l-rhamnose.

## Materials and Methods

### Materials

Glc-1-P, dTTP, and malachite green reagent were purchased from Sangon Biotech (Shanghai, China). Inorganic pyrophosphatase (YIPP) was purchased from Merck (Germany). D-glucosamine-1-phosphate (GlcNH_2_-1-P), 2-Azido-2-deoxy-d-glucose-1-phosphate (GlcN_3_-1-P), glucuronic acid-1-phosphate (GlcA-1-P), and N-acetylglucosamine-1-phosphate (GlcNAc-1-P) were kindly provided by Professor Junqiang Fang from the Shandong University, Qingdao. Ni^2+^ Sepharose high performance was purchased from GE Healthcare (Uppsala, Sweden). Other chemicals are analytical grade and are commercially available.

### Strains and Culture Conditions

The *S. syringae* CGMCC 4.1716 strain purchased from the China General Microbiological Culture Collection (CGMCC) Center was recovered and cultured in the 0234 broth (peptone 10g/l, yeast extract 2g/l, hydrolyzed casein 2g/l, NaCl 6g/l and glucose 10g/l, pH 7.2) as described by the supplier’s instruction. *E. coli* BL21 (DE3) used for protein expression was grown in Luria-Bertani (LB) medium at 37°C with 50μg/ml ampicillin (Sangon Biotech, China) added when required.

### Genes Cloning and Heterogeneous Expression

The four genes *rmlABCD* from *S. syringae* CGMCC 4.1716 were amplified with four pairs of specific primers (Supplementary Table S1) which were designed based on the target sequences (GenBank accession no. WP_033428542 for *rmlA*, WP_033429095 for *rmlB*, WP_033434852 for *rmlC*, and WP_033429096 for *rmlD*) using the genomic DNA of *S. syringae* CGMCC 4.1716 as PCR template. The purified PCR product was cloned into pET-22b plasmid with a histidine tag coding sequence fused to the C-terminal of each gene. The recombinant plasmid was transformed into *E. coli* BL21 (DE3) and the proper transformants were cultured in LB medium at 37°C. When the cell density reached 0.6–0.8 at 600nm, 0.25mM isopropyl β-d-thiogalactoside (IPTG) was added to the culture to induce the expression of the recombinant proteins. The cells were further cultured at 16°C for 16h, harvested by centrifugation, and lysed by ultrasonic treatment. The cell lysate was centrifuged, and the supernatant was used for protein purification with nickel affinity chromatography. The concentration of the purified proteins was determined using the Bradford method with bovine serum albumin as a standard.

### Molecular Mass Determination

The molecular masses of four Rml enzymes from *S. syringae* CGMCC 4.1716 (named Ss-RmlA, Ss-RmlB, Ss-RmlC, and Ss-RmlD) were determined by SDS-PAGE and gel filtration chromatography. SDS-PAGE was conducted with a 15% (w/v) gel, and proteins were visualized by Coomassie brilliant blue (CBB) R-250 staining. Gel filtration chromatography was carried out using a Superdex^™^ 200 increase gel filtration column (10×300, 8.6μm particle size, GE Healthcare, United States) on an AKTA purifier (GE Healthcare, United States). The standard proteins (GE Healthcare, United States) were aprotinin (6.5kDa), ribonuclease A (13.7kDa), carbonic anhydrase (29kDa) ovalbumin (43kDa), conalbumin (75kDa), aldolase (158kDa), ferritin (440kDa), and thyroglobulin (669kDa).

### Functional Confirmation of Ss-RmlABCD

The glucose-1-phosphate thymidylyltransferase activity of Ss-RmlA was confirmed by detecting the generation of dTDP-d-glucose from dTTP and Glc-1-P in a 100μl of reaction mixture containing 5.0mM dTTP, 5.0mM Glc-1-P, 10mM MgCl_2_, and 100μg/ml of Ss-RmlA in 40mM Tris–HCl buffer (pH 8.0). The dTDP-d-glucose 4,6-dehydratase activity of Ss-RmlB was confirmed by detecting the formation of dTDP-4-keto-6-deoxy-glucose in the Ss-RmlA reaction mixture supplemented with 5mM NAD^+^ and 100μg/ml of Ss-RmlB. The dTDP-4-keto-6-deoxy-glucose 3,5-epimerase activity of Ss-RmlC was confirmed by detecting the dTDP-4-keto-rhamnose formation in the Ss-RmlB reaction mixture supplemented with 100μg/ml of Ss-RmlC. The dTDP-4-keto-rhamnose reductase activity of Ss-RmlD was confirmed by detecting the dTDP-l-rhamnose formation in the Ss-RmlC reaction mixture supplemented with 5mM NADPH and 100μg/ml of Ss-RmlD. All the reactions were performed at 37°C for 5min and then terminated by mixing with 100μl chloroform. After centrifugation, the upper water phase was used for the detection of products by TLC and HPLC. The products were purified by HPLC and desalted for MS analysis.

### Enzyme Assays of Ss-RmlA and Ss-RmlB

The enzyme activity of Ss-RmlA was determined by measuring the amount of released PPi using a colorimetric method (Sha et al., 2011) with minor modifications. The 100μl reaction mixture containing 40mM Tris–HCl buffer (pH 8.0), 5.0mM dTTP, 5.0mM Glc-1-P, 10mM MgCl_2_, 100μg/ml of Ss-RmlA and 2U/ml YIPP was incubated at 37°C for 5min and terminated by mixing with 100μl malachite green reagent containing 0.03% (w/v) of malachite green, 0.2% (w/v) of ammonium molybdate, 0.05% (v/v) of Triton X-100, and 0.7N HCl. The absorbance of the mixture was measured at 630nm (OD_630_) with a microplate reader (BioTek, United States) after a 5-min incubation at 37°C. The amount of PPi released from the reaction was calculated using a standard curve relating OD_630_ value to PPi concentration. One unit of Ss-RmlA activity was defined as the amount of Ss-RmlA catalyzing the generation of 1μmol PPi per min under the assay conditions (Sha et al., 2011).

The enzyme activity of Ss-RmlB was determined by measuring the amount of generated dTDP-4-keto-6-deoxy-glucose using a colorimetric method (Shi et al., 2016) with minor modifications. The 100μl reaction mixture containing 40mM Tris–HCl buffer (pH 8.0), 5.0mM dTDP-d-glucose and 100μg/ml of Ss-RmlB was incubated at 37°C for 5min and terminated by mixing with 10μl of 1M NaOH solution at room temperature for 10min. Then the absorbance at 320nm (OD_320_) was detected with the microplate reader and the amount of dTDP-4-keto-6-deoxy-glucose was calculated using the standard curve of the OD_320_ value vs. dTDP-4-keto-6-deoxy-glucose concentration. One unit of Ss-RmlB activity was defined as the amount of Ss-RmlB catalyzing the generation of 1μmol dTDP-4-keto-6-deoxy-glucose per min under the assay conditions.

### Biochemical Studies of Ss-RmlA and Ss-RmlB

The optimal temperature for the reaction catalyzed by Ss-RmlA or Ss-RmlB was examined by assaying the enzyme activity at 16–80°C. Thermostability was determined by examining the residual enzyme activity after incubating the enzyme in the abovementioned temperature range for 1h. The optimal pH for the reaction was determined by assaying the enzyme activity at pH 3.5–12.0 in 40.0mM buffers (sodium acetate buffer at pH 3.5–6.5, Tris–HCl buffer at pH 7.5–9.0, and NaHCO_3_-NaOH buffer at pH 9.5–12.0). The pH stability was determined by incubating the enzyme in the abovementioned buffers at 4°C for 1h and assaying the residual enzyme activity. The effects of metal ions on Ss-RmlA activity were examined by assaying the enzyme activity in the presence of 2.0mM of EDTA, NaCl, KCl, ZnCl_2_, AgNO_3_, MnCl_2_, CaCl_2_, MgCl_2_, CuCl_2_, FeCl_2_, NiSO_4_, HgSO_4_, or CoSO_4_. The reaction without metal ions was used as the control. The optimal concentration of MgCl_2_ for Ss-RmlA activity was explored by assaying the enzyme activity in the presence of 0–5.5mM MgCl_2_. The optimal NAD^+^ concentration for Ss-RmlB activity was examined by assaying the enzyme activity in the presence of 0–0.08mM NAD^+^.

The kinetic analysis of Ss-RmlA for both substrates was conducted under the optimal reaction conditions using varied dTTP (0–0.5mM) and varied Glc-1-P (0–0.3mM) with the other one of these two substrates at a saturated concentration (1mM). The kinetic analysis of Ss-RmlB was conducted under the optimal reaction conditions using varied concentrations of dTDP-d-Glc (0–1.0mM). The *K*_m_ and *k*_cat_ values of the two enzymes were calculated using the software GraphPad Prism.6^1^

The substrate acceptance of Ss-RmlA for different NTPs and sugar-1-Ps was examined with a 100μl reaction mixture containing 2U/ml Ss-RmlA, 2U/ml YIPP, 2.5mM MgCl_2_, 5mM NTP, and 5mM sugar-1-P in 40mM NaHCO_3_-NaOH buffer (pH 9.5) at 37°C for 12h. The substrate dTTP was used to test other sugar-1-Ps (GlcNAc-1-P, GlcA-1-P, GlcNH_2_-1-P, GlcN_3_-1-P, and Man-1-P), and Glc-1-P was used to test other NTPs (ATP, dATP, GTP, dGTP, CTP, dCTP, UTP, and dUTP). Product formation was verified by MS analysis.

### One-Pot Synthesis of dTDP-l-Rhamnose and dUDP-l-Rhamnose

The synthesis was performed by simultaneously mixing all of the substrates, reagents, and four enzymes Ss-RmlABCD in one pot. To achieve the maximum yield, the reaction conditions including temperature, pH, NADPH concentration, enzyme concentration, and reaction time were evaluated. For the synthesis of dTDP-l-rhamnose, 10mM dTTP, 10mM Glc-1-P, 2.5mM MgCl_2_, 0.02mM NAD^+^, and 40mM Tris–HCl buffer were used. The effects of temperature (16–80°C) were determined using 5.0mM NADPH and 100μg/ml of each enzyme at pH 9.0. The effects of pH (3.5–12.0) were determined using 5.0mM NADPH and 100μg/ml of each enzyme at 30°C. The effects of NADPH concentration (0–8.0mM) were explored using 100μg/ml of each enzyme at pH 8.5 and 30°C. The effects of each enzyme concentration (100–300μg/ml) were determined using 1.5mM NADPH and 100μg/ml of each of the other three enzymes at pH 8.5 and 30°C. The effects of reaction time were evaluated by using 1.5mM NADPH, 100μg/ml of each of three enzymes (Ss-RmlA, Ss-RmlB, and Ss-RmlD) and 200μg/ml of Ss-RmlD at pH 8.5 and 30°C with interval sampling within 3h. For the synthesis of dUDP-l-rhamnose, except for 10mM dUTP, the components of the reaction mixture and conditions were the same as those for the synthesis of dUDP-l-rhamnose. All the reactions were performed for 20min and then terminated by mixing with an equal volume of chloroform. After centrifugation, the upper water phase was used for the detection of the product by TLC and HPLC. The products were purified by HPLC, and the identified fractions were concentrated by lyophilization. Then, the concentrated sample was desalted by being eluted from the Sephadex G10 column (10×300mm, GE Healthcare, United States) with distilled water at a flow rate of 1ml/min and detected at 260nm. The obtained pure product was lyophilized to dry powder and redissolved in distilled water and deuterated water for MS and nuclear magnetic resonance (NMR) analysis, respectively. The product yield was defined as the ratio of the concentration of the synthesized nucleotide-activated rhamnose (mM) to the concentration of added deoxynucleoside triphosphate (mM).

### TLC and HPLC Analysis

TLC was performed by loading samples on silica gel 60F254 plates (Merck, Germany). The loaded samples were developed by a mixture of 95% ethanol/1M acetic acid (5:2, pH 7.5; Kaminski and Eichler, 2014) and visualized by spraying the plate with 0.5% (w/v) 3,5-dihydroxytoluene in 20% (v/v) sulfuric acid and heating it at 120°C for 5min.

HPLC was performed on an Agilent 1,260 series HPLC system coupled with a UV detector (Agilent Technologies, Inc. United States) using the CarboPac^™^ PA-100 column (4×250mm, 4μm particle size, Thermo Fisher Scientific, United States). The sample was eluted with a gradient concentration of ammonium acetate, set as 0–30mM (0–12min), 30–60mM (12–22min), 100mM (22–32min), and 0 (32–37min), as the mobile phase at a flow rate of 1.0ml/min and detected at 260nm. The corresponding fractions were combined and concentrated through lyophilization.

### Mass Spectrometry and NMR

The mass spectra (MS) were recorded on a Shimadzu liquid chromatography-mass spectrometry ion trap time of flight (LCMS-IT-TOF) instrument (Kyoto, Japan) equipped with electrospray ionization (ESI) source in negative ion mode at a resolution of 10,000 full width at half-maximum. The nuclear magnetic resonance (NMR) spectra were recorded on an Agilent DD2 600MHz spectrometer (Agilent Technologies, Inc. United sTATES) at 600MHz for ^1^H and at 150MHz for ^13^C, and at 242MHz for ^31^P at 25°C. Chemical shifts were expressed in parts per million (ppm) downfield from the internal tetramethylsilane of D_2_O. Homo- and heteronuclear correlation experiments, including ^1^H−^1^H correlation spectroscopy (COSY), and heteronuclear single quantum coherence (HSQC) were run using the standard pulse sequences.

## Results

### Sequence Analysis and Expression of Ss-RmlA, Ss-RmlB, Ss-RmlC, and Ss-RmlD

**Figure 2:**
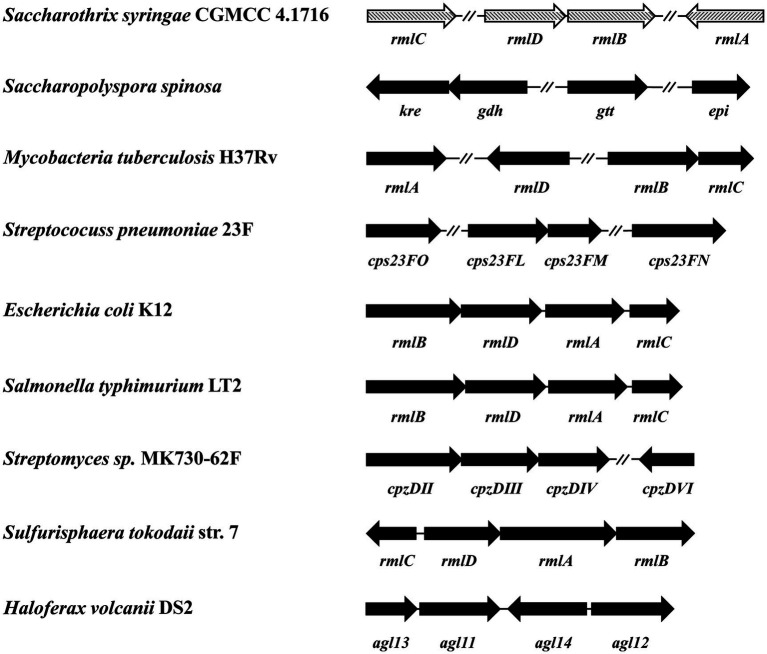
Comparison of nine sets of *rmlABCD* genes for genomic organization. The sequences for analysis were from *Saccharothrix syringae* CGMCC 4.1716, *Mycobacteria tuberculosis* H37Rv (Genbank accession no. NP_214848, NP_217981, NP_217982, and NP_217783)*, Saccharopolyspora spinosa* (Genbank accession no. AAK83289, AAK83290, AAK83291, and AAK83291), *Streptococcus pneumoniae* 23F (Genbank accession no. AAZ51352, WP_011285494, WP_012560640, and AAZ51220), *E. coli* K12 (Genbank accession no. AAB88400, AAB88398, AAB88401, and AAB88399), *Salmonella enterica* LT2 (Genbank accession no. CAA40117, CAA40115, CAA40118, and CAA40116), *Streptomyces sp*. MK730-62F (Genbank accession no. ADI50276, ADI50277, ADI50280, and ADI50278), *Sulfurisphaera tokodaii* str. 7 (Genbank accession no. BAK54697, BAB67067, BAB67064 and BAB67065), and *Haloferax volcanii* DS2 (Genbank accession no. ADE02576, ADE03524, ADE02686, and ADE02982). The genes were represented by arrows indicating their transcription orientation.

Four genes *Ss-rmlABCD* in the genome of *S. syringae* CGMCC 4.1716 are annotated to encode the putative dTDP-l-rhamnose synthetic pathway according to the GenBank database. The distribution pattern of these four genes in the genome of *S. syringae* CGMCC 4.1716 was compared with those of the homologs reported from four gram-positive bacteria (*Saccharopolyspora spinosa*, *Mycobacteria tuberculosis* H37Rv, *Streptomyces* sp. MK730-62F, and *Streptococcus pneumoniae* 23F), two gram-negative bacteria (*E. coli* K12 and *S. enterica* LT2), and two archaea (*Haloferax volcanii* DS2 and *Sulfurisphaera tokodaii* str. 7). The *rml* genes in the genome of *S. syringae* CGMCC 4.1716 were organized in three separate regions as *Ss-rmlC*, *Ss-rmlDB*, and *Ss-rmlA*, being different from those of the other homologs (Figure 2). The deduced amino acid sequences of the enzymes Ss-RmlA, Ss-RmlB, Ss-RmlC, and Ss-RmlD encoded by the *rml* genes from *S. syringae* CGMCC 4.1716 shared sequence identities of 33–81%, 46–80%, 35–56%, and 32–66% with those of the abovementioned homologs, respectively (Table 1).

**Table 1:** Overall homology of the predicted Ss-Rml enzymes from *Saccharothrix syringae* CGMCC 4.1716 with their homologs.

	Overall homology^a^ (%)			
	RmlA	RmlB	RmlC	RmlD
*S. syringae* CGMCC 4.1716	100/100	100/100	100/100	100/100
*Saccharopolyspora spinosa*	81/89	80/88	56/69	66/75
*Mycobacteria tuberculosis* H37Rv	59/74	48/63	40/54	53/62
*Streptomyces sp*. MK730-62F	38/57	72/77	47/64	53/61
*Streptococcus pneumoniae* 23F	62/79	44/63	30/45	35/51
*Escherichia coli* K12	61/76	45/59	33/46	35/50
*Salmonella typhimurium* LT2	60/76	46/60	36/50	36/50
*Haloferax volcanii* DS2	41/57	54/68	27/39	29/44
*Sulfurisphaera tokodaii* str. 7	33/55	46/59	35/52	32/50

a Overall homology is represented as percent identity/percent similarity.

**Figure 3:**
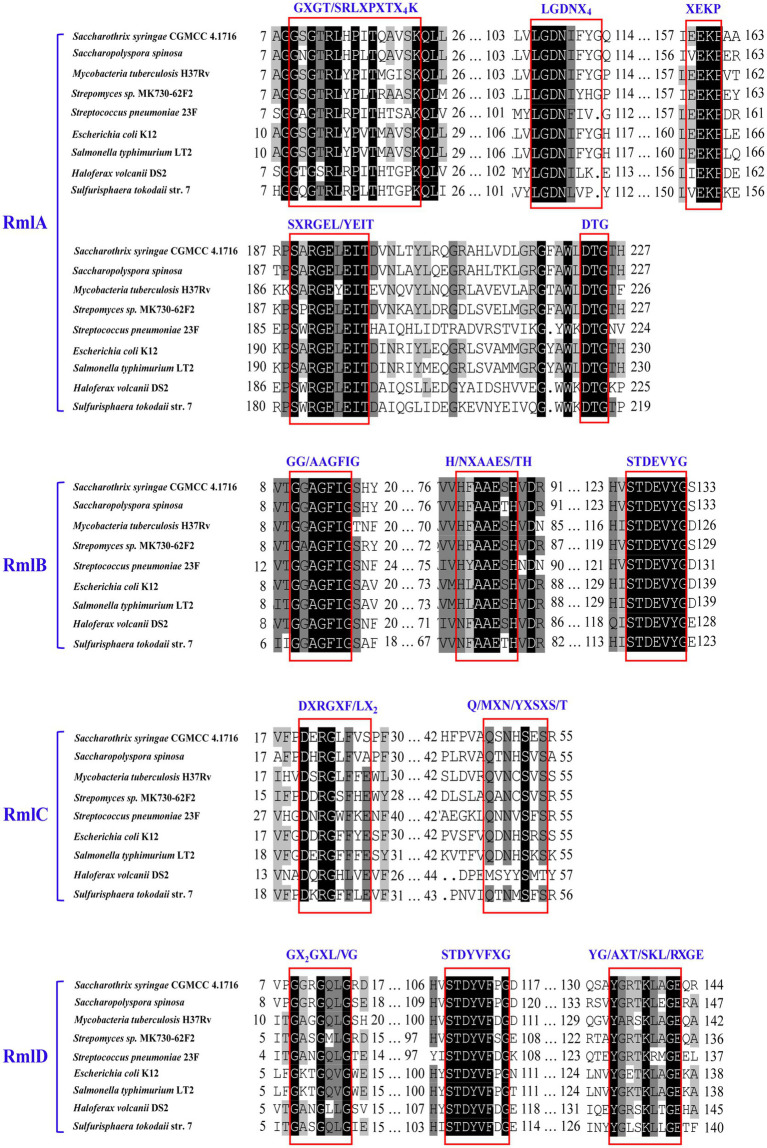
Multiple alignments of the partial amino acid sequences of RmlABCD. The sequences for alignment were from *Saccharothrix syringae* CGMCC 4.1716, *Saccharopolyspora spinosa*, *Mycobacteria tuberculosis* H37Rv, *Streptomyces sp.* MK730-62F, *Streptococcus pneumoniae* 23F, *E. coli* K12, *Salmonella typhimurium* LT2, *Haloferax volcani* DS2, and *Sulfurisphaera tokodaii* str. 7. The critical motifs are boxed and labeled.

The multiple alignments conducted with the predicted enzymes Ss-RmlABCD and their respective homologs from the abovementioned eight species indicated that there were several functionally critical motifs in the four Ss-Rml enzymes (Figure 3). Ss-RmlA possessed the motifs of GXGT/SRLXPXTX_4_K and LGDNX_4_ for the recognition and binding of dTTP, the motifs of XEKP and SXRGEXEIT for the recognition and binding of Glc-1-P, and Mg^2+^-stabilizing motifs of DTG and GDN within the LGDNX_4_. Ss-RmlB contained GG/AAGFIG, the signature motif of the short chain dehydrogenase/reductase (SDR) superfamily, as well as H/NXAAES/TH and STDEVYG, the motifs for recognition and binding of NAD^+^ and dTDP-glucose. Ss-RmlC had the substrate-binding motifs DXRGXF/LX_2_ and Q/MXN/YXSXS/T. Ss-RmlD possessed the GX_2_GX_2_G, a signature motif of reductases/epimerases/dehydrogenase superfamily, and the substrate-binding motifs STDYVFXG and YG/AXT/SKL/RXGE (Bais et al., 2018; Dhaked et al., 2019).

**Figure 4:**
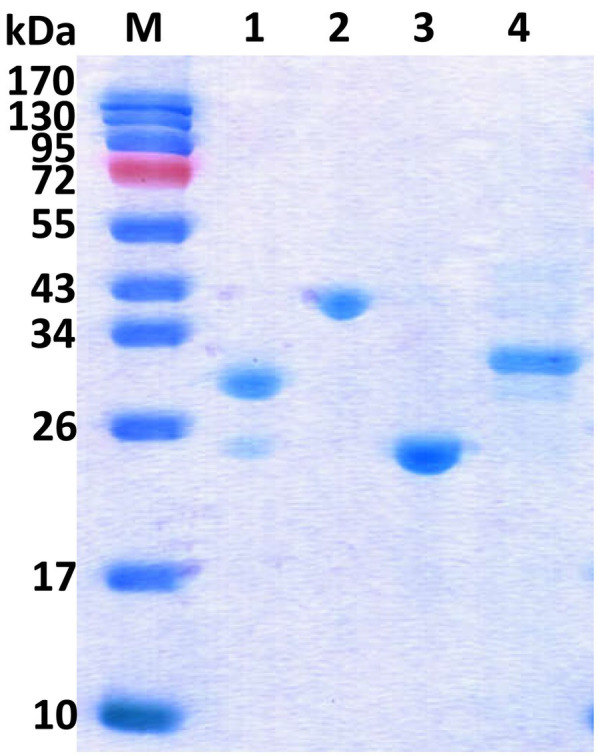
SDS-PAGE of the recombinant Rml enzymes from *S. syringae* CGMCC 4.1716. Lane M, protein marker; Lane 1, Ss-RmlA (32.7kDa); Lane 2, Ss-RmlB (37.2kDa); Lane 3, Ss-RmlC (23.1kDa); Lane 4, Ss-RmlD (32.4kDa). The SDS-PAGE was performed in a 15% (w/v) gel and the proteins were visualized by Coomassie brilliant blue R-250 staining.

The four *rmlABCD* genes from *S. syringae* CGMCC 4.1716 were then cloned from *S. syringae* CGMCC 4.1716 and successfully expressed in *E. coli* BL21 (DE3). As shown in the SDS-PAGE analysis (Figure 4), the recombinant proteins were purified to homogeneity. They showed the expected molecular masses of about 32.7kDa (Ss-RmlA), 37.2kDa (Ss-RmlB), 23.1kDa (Ss-RmlC), and 32.4kDa (Ss-RmlD) in agreement with the calculated masses fused with the histidine tag (about 1.0kDa). The result of gel filtration chromatography showed that the molecular masses of the four proteins in their native state were about 142.9kDa (Ss-RmlA), 87.6kDa (Ss-RmlB), 54.2kDa (Ss-RmlC), and 59.8kDa (Ss-RmlD), suggesting that Ss-RmlA was a homotetramer while Ss-RmlB, Ss-RmlC, and Ss-RmlD were homodimers.

### Functional Confirmation of Ss-RmlABCD

**Figure 5:**
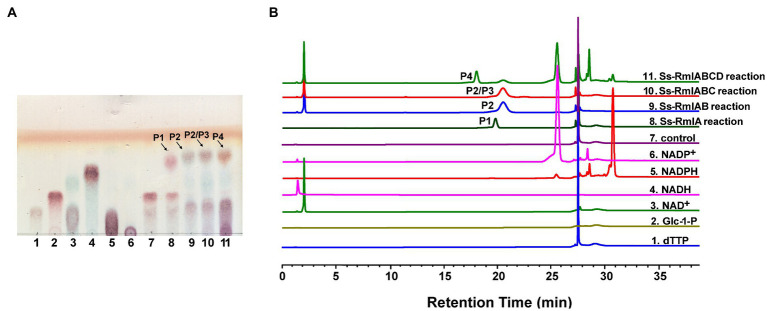
Analysis of the reactions catalyzed by Ss-RmlABCD through TLC **(A)** and HPLC **(B)**. **(A)** Lane 1, dTTP; Lane 2, Glc-1-P; Lane 3, NAD^+^; Lane 4, NADH; Lane 5, NADPH, Lane 6, NADP^+^, Lane 7, reaction control containing dTTP and Glc-1-P; Lane 8, reaction containing dTTP, Glc-1-P, and Ss-RmlA; Lane 9, reaction containing dTTP, Glc-1-P, NAD^+^, Ss-RmlA, and Ss-RmlB; Lane 10, reaction containing dTTP, Glc-1-P, NAD^+^, Ss-RmlA, Ss-RmlB, and Ss-RmlC; Lane 11, reaction containing dTTP, Glc-1-P, NAD^+^, NADPH, Ss-RmlA, Ss-RmlB, Ss-RmlC, and Ss-RmlD. The speculated products of Ss-RmlA, Ss-RmlB, Ss-RmlC, and Ss-RmlD are labeled as P1, P2, P3, and P4, respectively. **(B)** HPLC profile of the samples analyzed in **(A)** (Glc-1-P had no ultraviolet absorption under the analytical condition and thus could not be detected).

The enzyme activities of Ss-RmlA, Ss-RmlB, Ss-RmlC, and Ss-RmlD were analyzed by examining their catalytic products through TLC, HPLC, and MS. With dTTP and Glc-1-P as the substrates, Ss-RmlA catalyzed the formation of the product P1 in the reaction. As shown in Figure 5, a new spot of P1 appeared on the TLC plate and a peak with the retention time of 20min could be found in HPLC analysis. The negative-ion MS analysis of the product P1 showed the peak of [M-H]^−^ at *m/z* 563.0645, consistent with the theoretic molecular mass of dTDP-d-Glc (564.0758; Supplementary Figure S1). With the addition of NAD^+^ and Ss-RmlB in the Ss-RmlA reaction mixture, a new spot of product P2 appeared on the TLC plate and a peak with the retention time of 20.6min could be found in HPLC analysis (Figure 5). The product P2 was confirmed by MS as dTDP-4-keto-6-deoxy-Glc with the characteristic signal of [M-H]^−^ at *m/z* 545.0509 (Supplementary Figure S2). When Ss-RmlC was incubated in the reaction mixture of Ss-RmlA and Ss-RmlB, the spot of P3 on the TLC plate and its peak in HPLC were quite similar to those of P2 (Figure 5). Moreover, the substrate P2 (dTDP-4-keto-6-deoxy-Glc) and P3 (dTDP-4-keto-rhamnose) shared the identical molecular mass of 546.07, so the product P3 could not be confirmed here. After NADP^+^ and Ss-RmlD were incubated in the reaction mixture of three enzymes Ss-RmlABC, a new spot could be found on the TLC plate and a peak with the retention time of 18min could be recognized in HPLC analysis (Figure 5). And then P4 was confirmed by MS as dTDP-l-rhamnose with the characteristic signal of [M-H]^−^ at *m/z* 547.0770 (Supplementary Figure S11). The ^1^H-, ^13^C-, and ^31^P- NMR spectra of the product P4 (Supplementary Figure S12) agreed well with the published data of dTDP-l-rhamnose (Li et al., 2016). So, dTDP-l-rhamnose was successfully synthesized by the stepwise-catalysis of Ss-RmlABCD.

### Characterization of Ss-RmlA and Ss-RmlB

**Figure 6:**
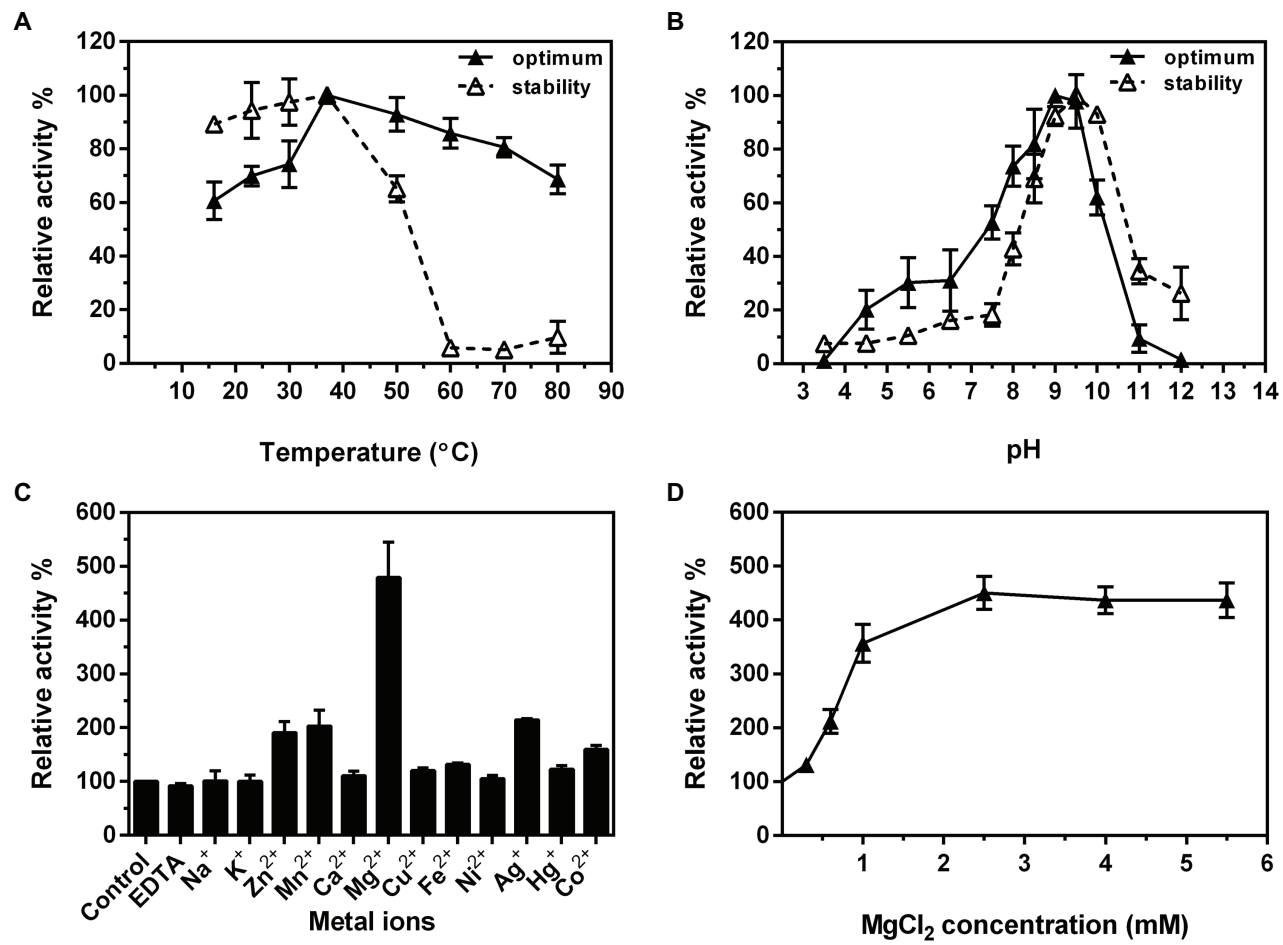
Effects of reaction temperature **(A)** and pH **(B)** on the enzyme activity and stability of Ss-RmlA. Effects of metal ions **(C)** and concentration of MgCl_2_ **(D)** on the enzyme activity of Ss-RmlA. Data points represent the means ± S.D. of three replicates.

After confirming the functions of the four Rml enzymes from *S. syringae* CGMCC 4.1716, we further characterized Ss-RmlA and Ss-RmlB, the first two enzymes of the pathway. Ss-RmlA exhibited the maximal activity at 37°C and was stable below 37°C (Figure 6A). The enzyme was highly active in the pH range of 8.5–9.5 with the maximal activity obtained at pH 9.0, and it was stable at pH 9.0–10.0 (Figure 6B). Mg^2+^ exhibited the strongest promoting effect on the activity of Ss-RmlA, increasing the activity by 4.8 times. Zn^2+^, Mn^2+^, Ag^2+^, and Co^2+^ promoted the enzyme activity by 1.9, 2.0, 2.1, and 1.6 times, respectively. The other tested ions Na^+^, K^+^, Ca^2+^, Cu^2+^, Fe^2+^, Ni^2+^, Hg^2+^, and EDTA hardly affected the enzyme activity (Figure 6C). The optimal Mg^2+^ concentration for Ss-RmlA activity was determined to be 2.5mM (Figure 6D). The *K*_m_ and *k*_cat_ values of Ss-RmlA for dTTP and Glc-1-P were 49.56μM and 5.39s^−1^, and 117.30μM and 3.46s^−1^, respectively.

The substrate specificity of Ss-RmlA was then analyzed. In addition to dTTP and Glc-1-P, eight NTPs (ATP, dATP, GTP, dGTP, CTP, dCTP, UTP, and dUTP) and five sugar-1-Ps (GlcNAc-1-P, GlcA-1-P, GlcNH_2_-1-P, GlcN_3_-1-P, and Man-1-P) were tested as the substrates. Among them, three NTPs (dTTP, dUTP, and UTP) and three sugar-1-Ps (Glc-1-P, GlcNH_2_-1-P, and GlcN_3_-1-P) were found to be used by Ss-RmlA, forming a total of nine NDP-sugars. In addition to dTDP-Glc, the other eight products were identified by MS to be dTDP-GlcNH_2_, dTDP-GlcN_3_, UDP-Glc, UDP-GlcNH_2_, UDP-GlcN_3_, dUDP-Glc, dUDP-GlcNH_2_, and dUDP-GlcN_3_ (Table 2 and Supplementary Figures S3–S10).

**Table 2:** MS analysis of the products catalyzed by Ss-RmlA.

Compound	Calculated [M-H]*^−^m/z^-^*	Found [M-H]*^−^m/z^-^*
dTDP-Glc	563.0679	563.0645
dTDP-GlcNH_2_	562.0839	562.0825
dTDP-GlcN_3_	588.0744	588.0708
UDP-Glc	565.0472	565.0432
UDP-GlcNH_2_	564.0632	564.0587
UDP-GlcN_3_	590.0537	590.0494
dUDP-Glc	549.0523	549.0509
dUDP-GlcNH_2_	548.0683	548.0671
dUDP-GlcN_3_	574.0588	574.0573

**Figure 7:**
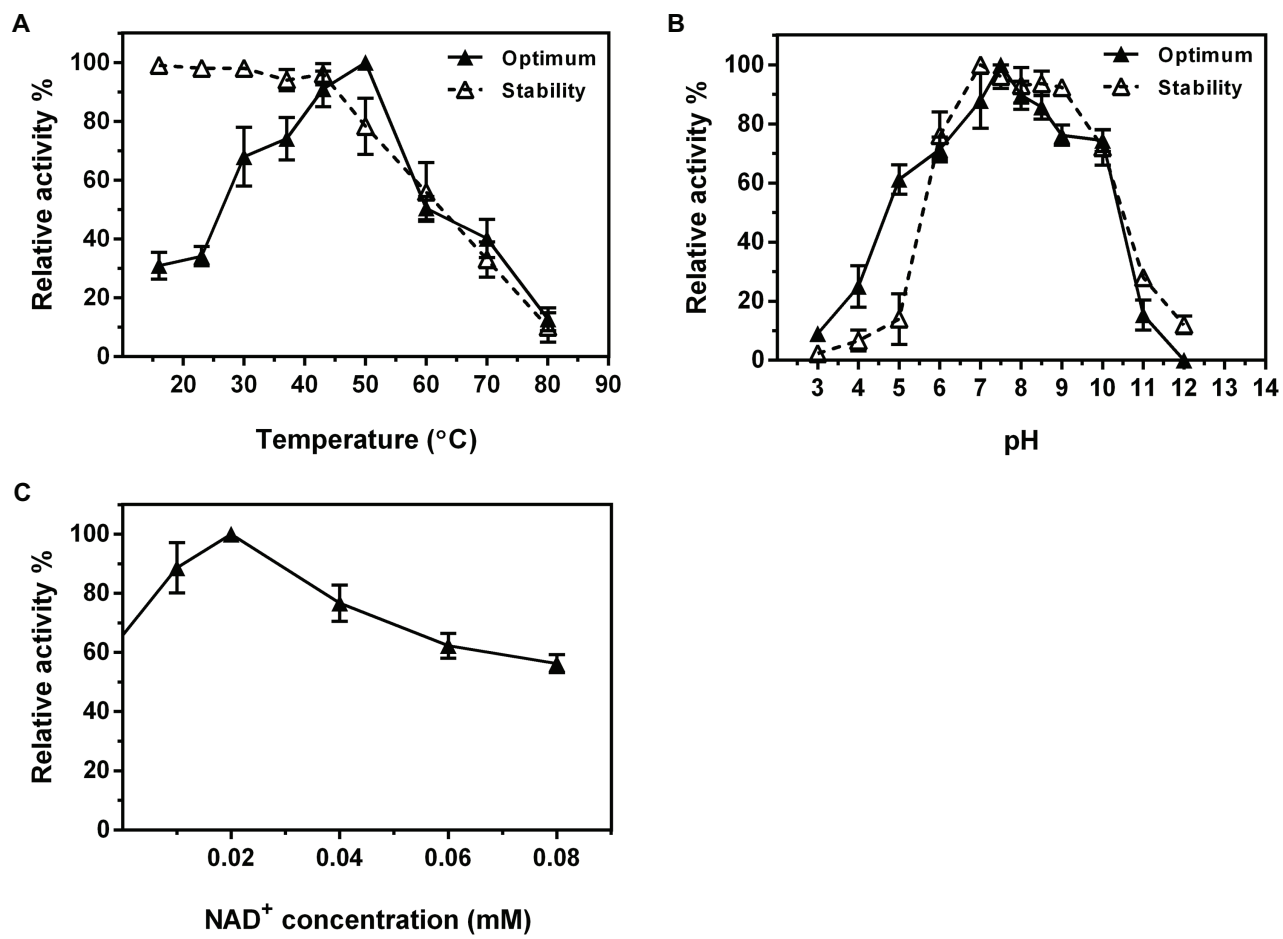
Effects of reaction temperature **(A)** and pH **(B)** on the enzyme activity and stability of Ss-RmlB. Effect of NAD^+^ concentration on the enzyme activity of Ss-RmlB **(C)**. Data points represent the means ± S.D. of three replicates.

The optimal temperature for Ss-RmlB activity was 50°C, and the enzyme was stable below 42°C (Figure 7A). Ss-RmlB was highly active in the pH range of 7.0–8.5 with the maximal activity obtained at pH 7.5, and the enzyme was stable at pH 7.0–9.0 (Figure 7B). NAD^+^ could promote Ss-RmlB activity, and the optimal concentration of NAD^+^ was 0.02mM. When NAD^+^ exceeded 0.02mM, the enzyme activity dropped considerably (Figure 7C). The *K*_m_ and *k*_cat_ values of Ss-RmlB for dTDP-glucose were 98.60μM and 11.2s^−1^, respectively.

### One-Pot Synthesis of dTDP-l-Rhamnose and dUDP-l-Rhamnose

**Figure 8:**
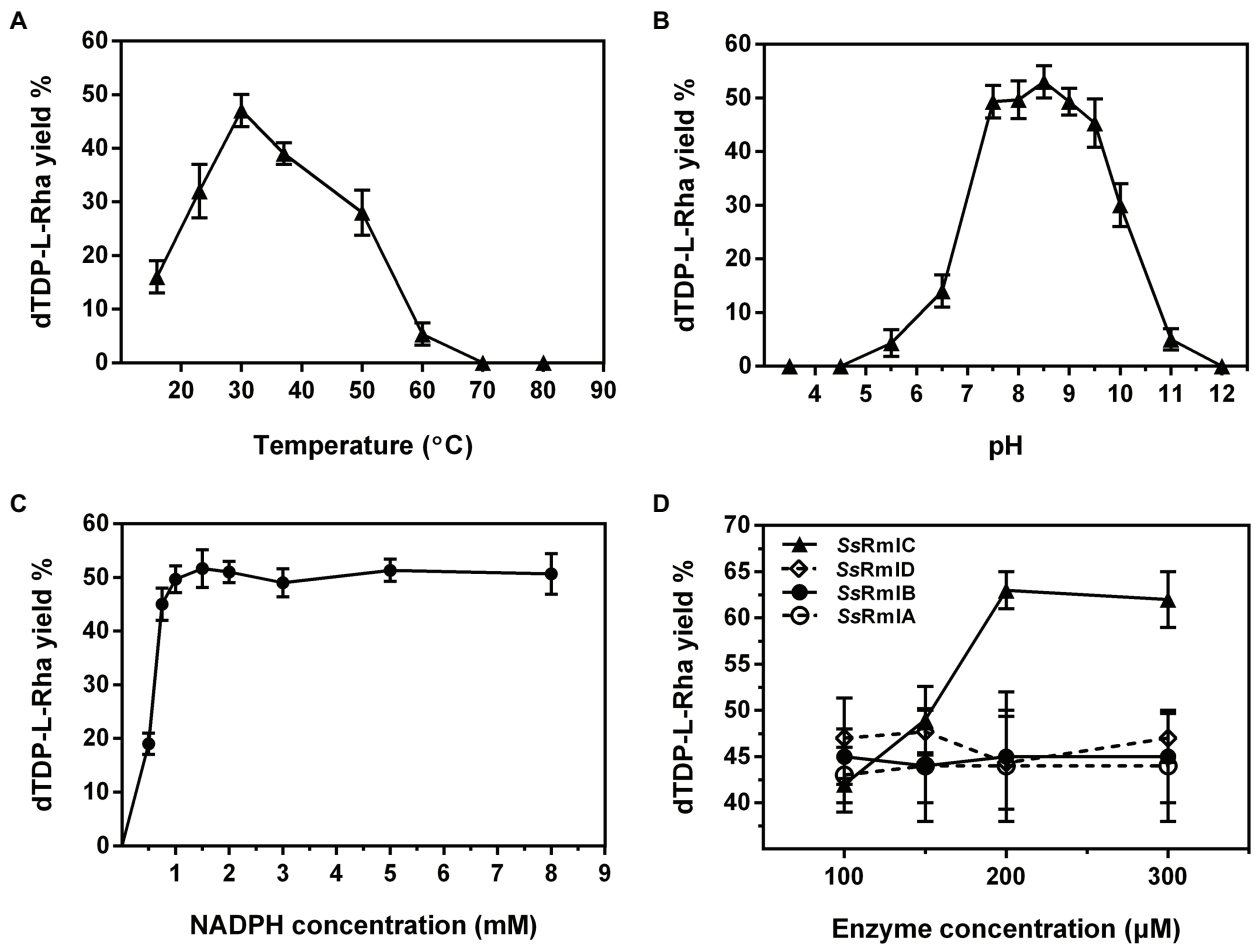
The effects of temperature **(A)**, pH **(B)**, NADPH concentration **(C)**, and enzyme concentration **(D)** on the yield of dTDP-l-rhamnose catalyzed by Ss-RmlABCD. Data points represent the means ± S.D. of three replicates.

**Figure 9:**
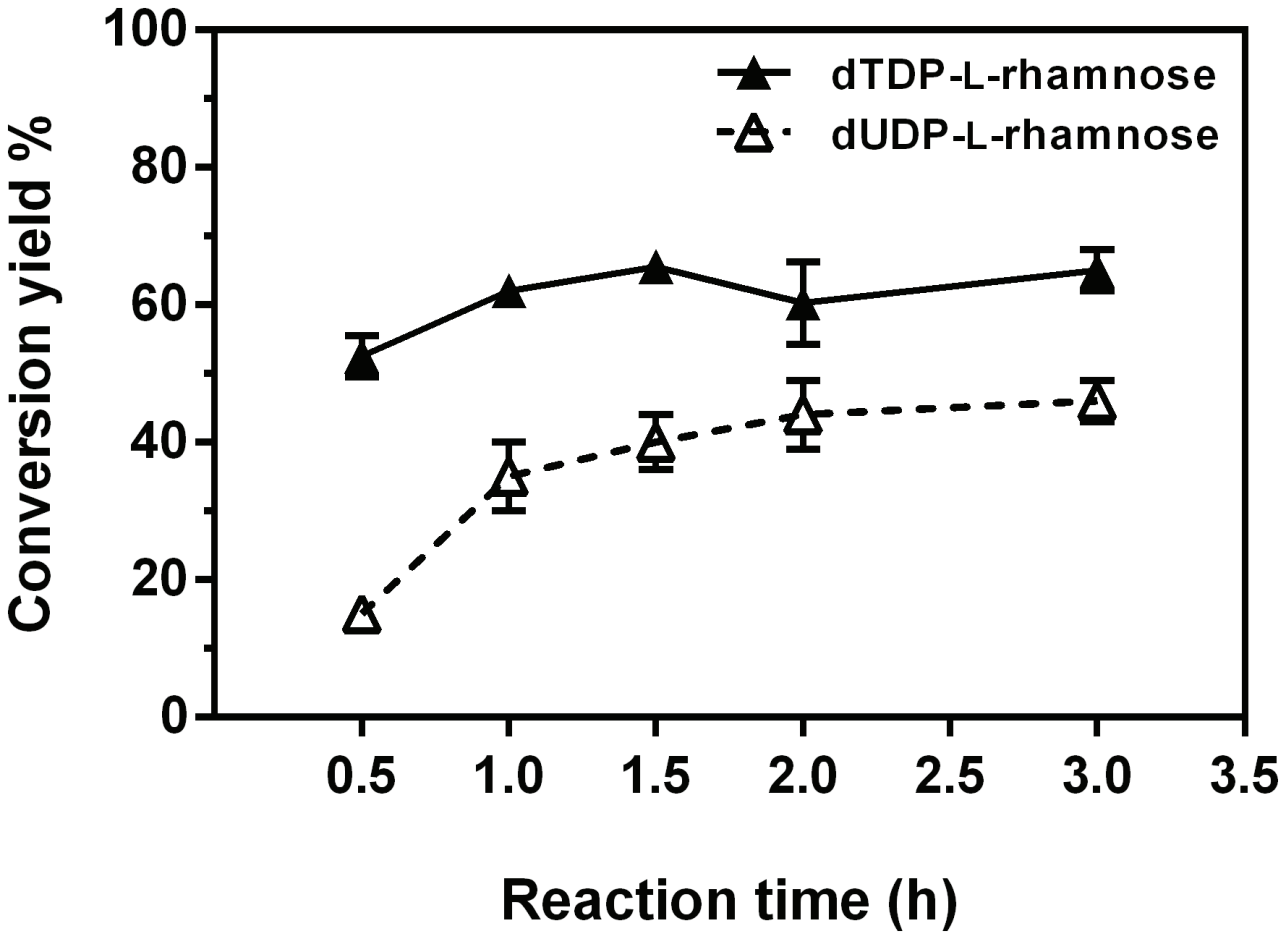
Time course of one-pot synthesis of dTDP-l-rhamnose and dUDP-l-rhamnose under the optimal reaction conditions.

The one-pot synthesis of dTDP-l-rhamnose was performed by incubating four enzymes Ss-RmlABCD with dTTP and Glc-1-P as the starting substrates. The effects of temperature, pH, and concentrations of NADPH and enzymes on dTDP-l-rhamnose yield were investigated in detail. As shown in Figure 8A, the reaction temperature markedly affected dTDP-l-rhamnose formation. As the temperature was raised from 16 to 70°C, dTDP-l-rhamnose yield rapidly increased from 14% to the maximum of 47% at 30°C, decreased to 28% at 50°C, and dropped to 0 at 70°C. Thus, subsequent reactions were performed at 30°C. The pH values also strongly affected dTDP-l-rhamnose yield. Figure 8B showed that dTDP-l-rhamnose yield increased at pH 3.5–7.5 and then stabilized at pH 7.5–9.5 with the maximal yield of 53% obtained at pH 8.5. When pH exceeded 9.5, dTDP-l-rhamnose yield sharply decreased to 0 at pH 12.0. Thus, the subsequent reactions were performed at pH 8.5. NADPH was an essential cofactor for the final step of reduction catalyzed by Ss-RmlD and the effect of its concentration on dTDP-l-rhamnose yield was examined. As shown in Figure 8C, when NADPH was increased from 0 to 1.5mM, dTDP-l-rhamnose yield increased from 0 to the maximum of 52% and then kept stable at 1.5–8.0mM. Thus, the subsequent reactions were performed using 1.5mM of NADPH. Figure 8D showed the effect of each enzyme concentration on dTDP-l-rhamnose yield. When the concentration of each enzyme changed from 100 to 300μg/ml, the change of the concentration of Ss-RmlC from 100 to 200μg/ml significantly influenced dTDP-l-rhamnose yield with a significant increase from 42 to 63%, but the change of the concentration of each of three enzymes Ss-RmlA, Ss-RmlB, and Ss-RmlD did not affect the dTDP-l-rhamnose yield. Therefore, the optimal conditions for one-pot synthesis of dTDP-l-rhamnose was 10mM dTTP, 10mM Glc-1-P, 0.02mM NAD^+^, 1.5mM NADPH, 100μg/ml of each of three enzymes Ss-RmlABD, and 200μg/ml of Ss-RmlC at pH 8.5 and 30°C. The time curves indicated that dTDP-l-rhamnose yield reached the maximum of 65% at 90min (Figure 9).

Next, the positive substrates of Ss-RmlA, including dTTP, dUTP, UTP, Glc-1-P, GlcNH_2_-1-P, and GlcN_3_-1-P were tested as the starting substrates in a one-pot reaction under the optimal synthesis conditions for dTDP-l-rhamnose. In addition to dTTP and Glc-1-P, dUTP and Glc-1-P could also be used by Ss-RmlABCD as the starting substrates to form a new product with a yield of 46% at 90min (Figure 9). This product was purified by HPLC, and identified by MS with the peak of [M-H]^−^ at *m/z* 533.0538 in the negative ion ESI mass analysis, consistent with the theoretical molecular mass of dUDP-l-rhamnose (534.0652; Supplementary Figure S13). The chemical structure of this new product was further elucidated by ^1^H, ^13^C, ^31^P, ^1^H-^1^H COSY, ^1^H-^13^C HSQC, and ^1^H-^13^C HSQC without decoupling NMR analysis (Supplementary Figures S14–S19). The proton signal of the peak at δ 5.04ppm and the carbon signal of the peak at δ 95.4ppm in the ^1^H- and ^13^C-NMR spectra were, respectively, assigned to be the H-1 and C-1 of the rhamnose moiety (Supplementary Figures S14 and S15). According to the ^1^H-^13^C HSQC spectrum without decoupling (Supplementary Figure S19), the coupling constant of the C-1 and H-1 (*J*_C1, H1_) of the rhamnose moiety was determined to be 162Hz, revealing a β-configuration of its anomeric carbon. Therefore, the product was identified as dUDP-β-l-rhamnose.

## Discussion

Bacterial rhamnosyltransferases are advantageous biocatalysts for the synthesis of rhamnose-containing biomolecules due to their high catalytic efficiency and flexible glycosyl acceptor specificity. But their application is limited by the availability of the glycosyl donor dTDP-l-rhamnose which is difficult to achieve through chemical synthesis. In bacteria and archaea, dTDP-l-rhamnose is synthesized by four conserved enzymes RmlABCD. Thus far, some studies of enzymatic synthesis of dTDP-l-rhamnose *in vitro* with a set of bacterial RmlABCD or partial bacterial Rml enzymes combined with other enzymes from potato and *Saccharomyces cerevisiae* have been reported (Marumo et al., 1992; Pazur and Shuey, 2002; Oh et al., 2003; Elling et al., 2005; Kang et al., 2006; Li et al., 2016). The discovery of a novel set of RmlABCD enzymes and the development of a simple and efficient enzymatic synthesis reaction system would be very worthy of further exploration. In this work, a novel set of four novel Ss-RmlABCD enzymes from *S. syringae* CGMCC 4.1716 were heterologously expressed and functionally confirmed, and one-pot synthesis of dTDP-l-rhamnose (yield of 65%) and dUDP-l-rhamnose (yield of 46%) by employing Ss-RmlABCD enzymes was developed.

The genomic organizations of *rmlABCD* genes in prokaryotic microorganisms are polymorphic. The *rmlABCD* genes of *E. coli* K12, *S. enterica* LT2, *Sulfurisphaera tokodaii* str. 7, *Haloferax volcanii* DS2, and *Streptomyces* sp. MK730-62F are successively located within the biosynthetic gene clusters for cell wall glycans or the secondary metabolite. On the contrary, for some other species, the *rmlABCD* genes are separately located on their genomes, spaced apart by other genes. For example, the four genes were arranged in three regions in the order of *rmlDB*, *rmlA*, and *rmlC* in *Saccharopolyspora spinosa*, *rmlA*, *rmlD*, and *rmlBC* in *Mycobacteria tuberculosis* H37Rv, as well as *rmlD*, *rmlAC*, and *rmlB* in *Streptococcus pneumoniae* 23F. In this study, the four *Ss-rmlABCD* genes from *S. syringae* CGMCC 4.1716 were found to be also located in three discrete regions of the genome in the order of *Ss-rmlC, Ss-rmlDB*, and *Ss-rmlA*, presenting a different pattern compared with those of the reported homologs.

Among four Rml enzymes, the enzyme RmlA is the most reported (Han et al., 2007; Alphey et al., 2013; Brown et al., 2017). The RmlA from other bacteria usually showed maximal enzyme activities at pH 7.0–8.5 and an absolute requirement for divalent metal ions, particularly Mg^2+^ (Zuccotti et al., 2001; Graninger et al., 2002; Sha et al., 2011; Li et al., 2016). However, in this study, Ss-RmlA was maximally active in the pH range of 8.5–9.5 and stable in the pH range of 9.0–10.0. Ss-RmlA could exhibit activity when metal ions were absent but the addition of 2.5mMMg^2+^ showed the maximal promoting effect on its activity, increasing the activity by 4.8 times. It was worth noting that Zn^2+^, Mn^2+^, Ag^+^, and Co^2+^ could weakly promote Ss-RmlA activity, which was not common for the other reported RmlA homologs.

Sugar nucleotidylyltransferases are a family of enzymes catalyzing the formation of sugar nucleotides from NTPs and sugar-1-Ps (Mizanur et al., 2004; Bais et al., 2018; Li et al., 2020). Based on the variation of the motifs for stabilization of Mg_A_^2+^, one of the two magnesium ions required in catalysis, these enzymes can be classified into five groups: IA, IB, IC, IIA, and IIB. Most sugar nucleotidylyltransferases with the group IC-specific Mg_A_^2+^-stabilizing motifs have been reported to be promiscuous for substrates (Zhang et al., 2005; Bais et al., 2018). For example, the Glc-1-P thymidylyltransferase PH^GT^ from *Pyrococcus horikoshii* could use UTP, CTP, GTP, dTTP, Glc-1-P, and glucosamine-1-phosphate (GlcN-1-P) as substrates (Bais et al., 2018). The Glc-1-P uridyltransferase ST^GU^ from *Streptococcus thermophilus* could also use UTP, dTTP, GTP, CTP, Glc-1-P, Man-1-P, GlcN-1-P, and GlcNAc-1-P as substrates (Bais et al., 2018). In this work, Ss-RmlA was found to possess the conserved motifs GDN (residues 107–109) and DTG (residues 223–225), which were the Mg_A_^2+^-stabilizing motifs peculiar to the sugar nucleotidylyltransferases of group IC. This finding was consistent with the result that Ss-RmlA used three NTPs (dTTP, dUTP, and UTP) and three sugar-1-Ps (Glc-1-P, GlcNH_2_-1-P, and GlcN_3_-1-P) as substrates to produce nine NDP-sugars, including dTDP-Glc, dTDP-GlcNH_2_, dTDP-GlcN_3_, UDP-Glc, UDP-GlcNH_2_, UDP-GlcN_3_, dUDP-Glc, dUDP-GlcNH_2_, and dUDP-GlcN_3_, indicating that Ss-RmlA was a sugar nucleotidylyltransferase of group IC.

Although RmlABCD enzymes act in synergy to synthesize dTDP-l-rhamnose, some individual Rml enzymes show different optimal reaction conditions compared with other enzymes in the same pathway. The variance was observed for both bacterial and archaeal Rml homologs. For example, Cps23FL (RmlA) and Cps23FN (RmlB) from *Streptococcus pneumonia* serotype 23F shared the same optimal pH for enzyme activity, but the optimal temperature for Cps23FN (37°C) was 12°C higher than that of Cps23FL (25°C; Li et al., 2016). For the RmlABCD enzymes from *Sulfolobus tokodaii* strain 7, RmlA showed maximal activity at pH 8.5 and 95°C, whereas RmlB was most active at 80°C (Zhang et al., 2005; Teramoto et al., 2012). In our study, Ss-RmlA displayed maximal enzyme activity at pH 9.0 and 37°C, while Ss-RmlB showed maximal enzyme activity at pH 7.5 and 50°C. Thus, in order to maximize dTDP-l-rhamnose yield, we optimized the conditions for the one-pot reaction catalyzed by Ss-RmlABCD. The optimal pH and temperature determined for the one-pot reaction were pH 8.5 and 30°C which were different from those of Ss-RmlA and Ss-RmlB, suggesting that the optimal conditions for the one-pot four-enzyme reaction could be a compromise for those of the four enzymes.

After the optimization of conditions including pH, temperature, and NADPH concentration for the one-pot reaction, we next analyzed the effect of enzyme concentration on dTDP-l-rhamnose production. As shown in Figure 8D, the increase of Ss-RmlC concentration from 100 to 200μg/ml led to a corresponding rise in dTDP-l-rhamnose yield from 42 to 63%, but the concentration change from 100 to 300μg/ml of Ss-RmlA, Ss-RmlB or Ss-RmlC could not lead to a similar effect, which suggested that these three enzymes might be surplus for catalysis even at the concentration of 100μg/ml.

The one-pot enzymatic synthesis of dTDP-l-rhamnose by RmlABCD involves multiple factors possibly limiting the product yield. RmlA, the first enzyme in the pathway, was reported to be significantly inhibited by the end product dTDP-l-rhamnose *in vitro*, which in turn limited the final yield. Li et al. reported that quite a low yield (~1%) of dTDP-l-rhamnose was obtained when Cps23FL, Cps23FN, Cps23FM, and Cps23FO were simultaneously used in one pot. On the contrary, the dTDP-l-rhamnose yield could be improved to 63% when the four enzymes were added in two portions, in which the first enzyme Cps23FL was added to synthesize dTDP-d-glucose firstly, and then the enzymes Cps23FN, Cps23FM, and Cps23FO (RmlBCD) were supplemented to the reaction mixture to yield dTDP-l-rhamnose (Li et al., 2016). With such an operation, they minimized the inhibition of dTDP-l-rhamnose on Cps23FL (RmlA) activity. In contrast, we did not observe the obvious inhibitory effect of dTDP-l-rhamnose on Ss-RmlA activity and obtained a similar yield of dTDP-l-rhamnose whether we added Ss-RmlABCD simultaneously or in two portions as Li et al. reported (data not shown), suggesting that dTDP-l-rhamnose probably had no significant inhibitory effect on the enzyme activity of Ss-RmlA.

Several sets of Rml enzymes from different bacterial species have been successfully employed to synthesize dTDP-l-rhamnose *in vitro*. The highest yield of dTDP-l-rhamnose reported so far is 63% achieved by Li et al. using four Rml homologs Cps23FL, Cps23FN, Cps23FM, and Cps23FO from *S*. *pneumonia* serotype 23F (Li et al., 2016). However, the authors here had to carry out the synthesis reaction by adding the four involved enzymes in two portions in order to avoid the inhibition on Cps23FL by dTDP-l-rhamnose. In this work, by simultaneously mixing Ss-RmlABCD enzymes, the substrates, and the necessary reagents in one pot, we developed a simple four-enzyme reaction system for the synthesis of dTDP-l-rhamnose with a comparable conversion yield (65%). Moreover, using this reaction system, we successfully synthesized a structural analog of dTDP-l-rhamnose, dUDP-l-rhamnose, which was an unnatural nucleotide-activated rhamnose reported for the first time. Next, in order to develop a more cost-effective one-pot enzymatic process for the synthesis of dTDP-l-rhamnose or dUDP-l-rhamnose, enzyme immobilization and cell surface display techniques could be strategies worthy of attempts to reduce the cost of enzymes (Zhang et al., 2006; Silva-Salinas et al., 2021).

In conclusion, this work identified and characterized a novel set of dTDP-l-rhamnose synthetic enzymes Ss-RmlABCD from *S. syringae* CGMCC 4.1716 and provided a new simple and efficient reaction system that laid a foundation for the practical enzymatic synthesis of dTDP-l-rhamnose and dUDP-l-rhamnose.

## Data Availability Statement

The datasets presented in this study can be found in online repositories. The names of the repository/repositories and accession number(s) can be found in the article/Supplementary Material.

## Author Contributions

SY conceived and designed the research and wrote the manuscript. XA and SY performed the experiments and analyzed the data. SY, LX, and MX revised the manuscript. LX, XJ and MX supervised the project. All authors contributed to the article and approved the submitted version.

## Funding

This work was partly supported by National Key Research and Development Program of China (2018YFA0902000), the National Natural Science Foundation of China (31872626), and Central Government Guide Local Science and Technology Development Funds (YDZX20203700002579).

## Conflict of Interest

The authors declare that the research was conducted in the absence of any commercial or financial relationships that could be constructed as a potential conflict of interest.

## Publisher’s Note

All claims expressed in this article are solely those of the authors and do not necessarily represent those of their affiliated organizations, or those of the publisher, the editors and the reviewers. Any product that may be evaluated in this article, or claim that may be made by its manufacturer, is not guaranteed or endorsed by the publisher.

## Footnotes

^1^ http://www.graphpad.com/scientificsoftware/prism/
