# Supplementary material for: Novel dTDP-l-Rhamnose Synthetic Enzymes (RmlABCD) From Saccharothrix syringae CGMCC 4.1716 for One-Pot Four-Enzyme Synthesis of dTDP-l-Rhamnose
Source: Front Microbiol. 2021 Nov 8;12:772839. doi: 10.3389/fmicb.2021.772839 (PMC8606822; doi:10.3389/fmicb.2021.772839)
Supplement: Supplementary file 1 [file Data_Sheet_1.PDF]

Supplemental Material

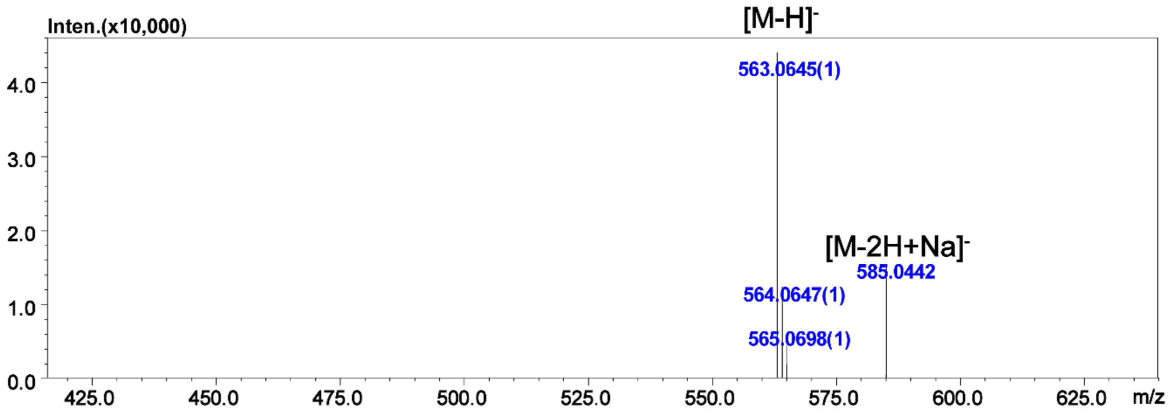

Supplementary Figure S1. HR-ITTOF-ESI/MS analysis of dTDP-D-Glc (M_w 564.0758) synthesized by Ss-RmlA.

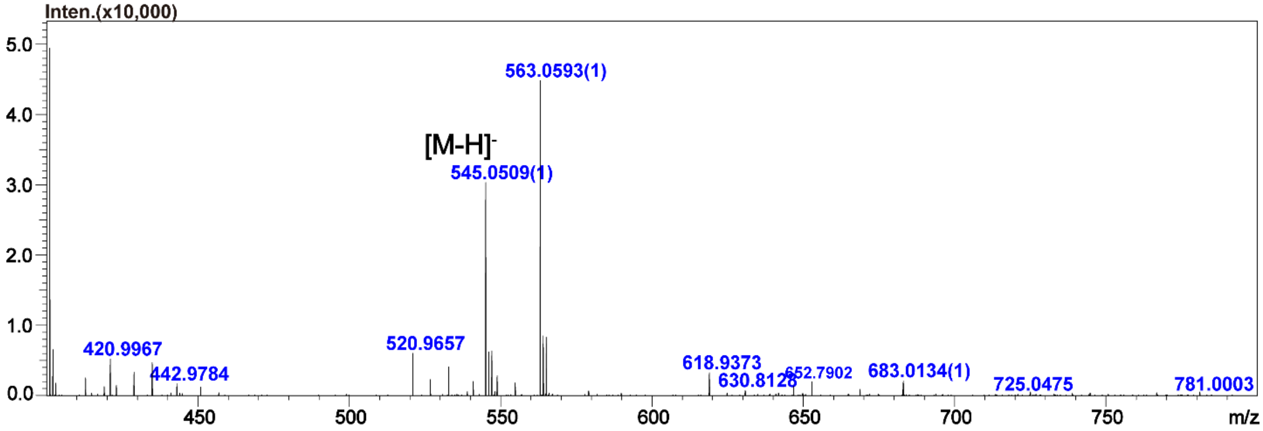

Supplementary Figure S2. HR-ITTOF-ESI/MS analysis of dTDP-4-keto-6-deoxy-Glc (M_w 546.07) synthesized by Ss-RmlA.

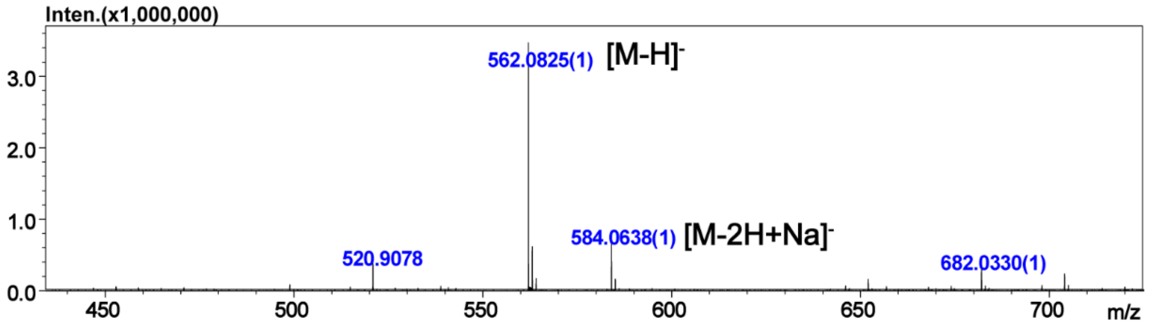

Supplementary Figure S3. HR-ITTOF-ESI/MS analysis of dTDP-D-GlcNH$_2$ (M_w 563.0917) synthesized by Ss-RmlA.

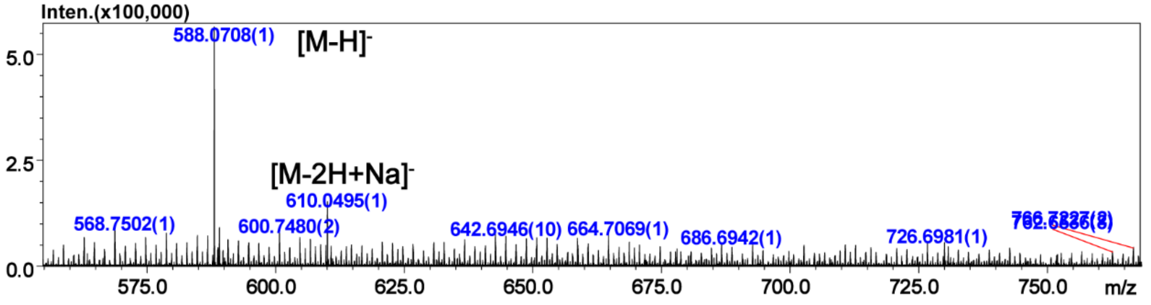

Supplementary Figure S4. HR-ITTOF-ESI/MS analysis of dTDP-D-GlcN$_3$ (M_w 589.0822) synthesized by Ss-RmlA.

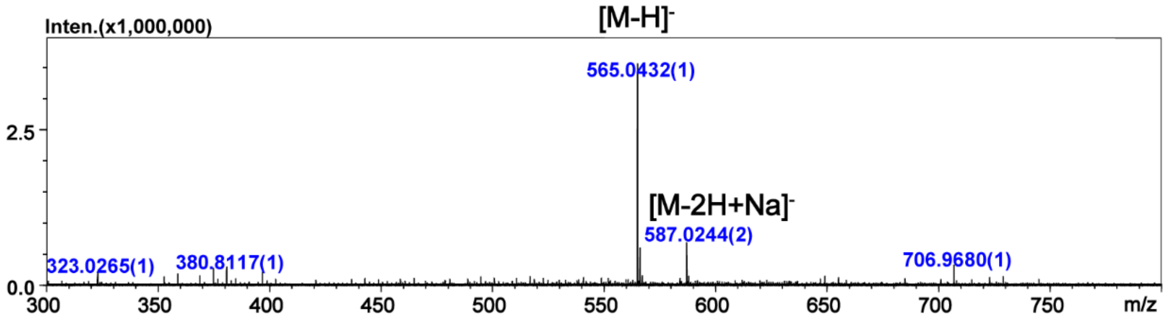

Supplementary Figure S5. HR-ITTOF-ESI/MS analysis of UDP-D-Glc (M_w 566.0550) synthesized by Ss-RmlA.

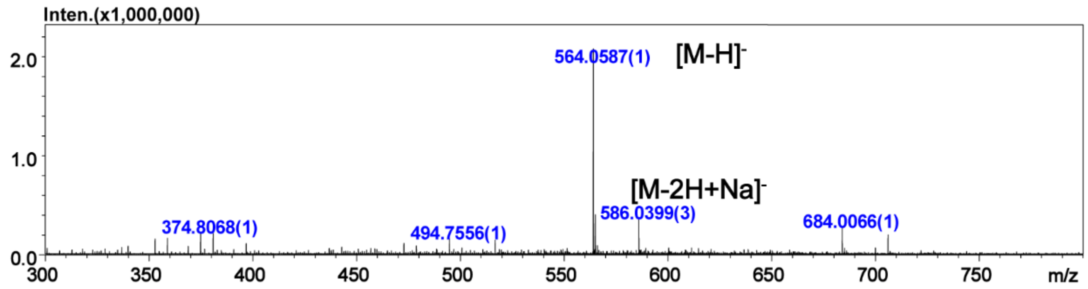

Supplementary Figure S6. HR-ITTOF-ESI/MS analysis of UDP-D-GlcNH_2 (M_w 565.0710) synthesized by Ss-RmlA.

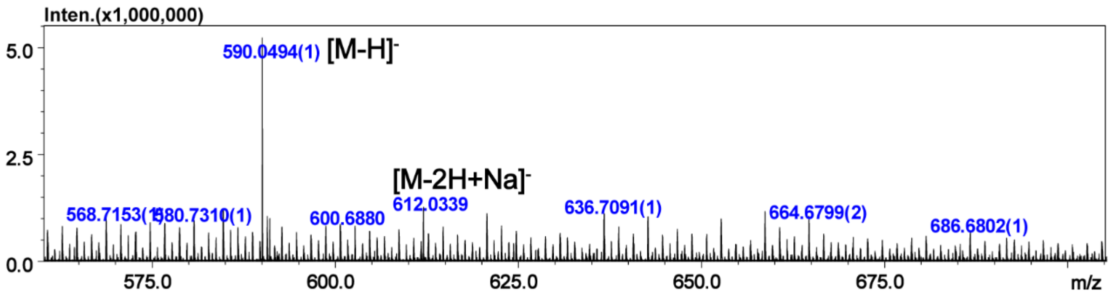

Supplementary Figure S7. HR-ITTOF-ESI/MS analysis of UDP-D-GlcN_3 (M_w 591.0615) synthesized by Ss-RmlA.

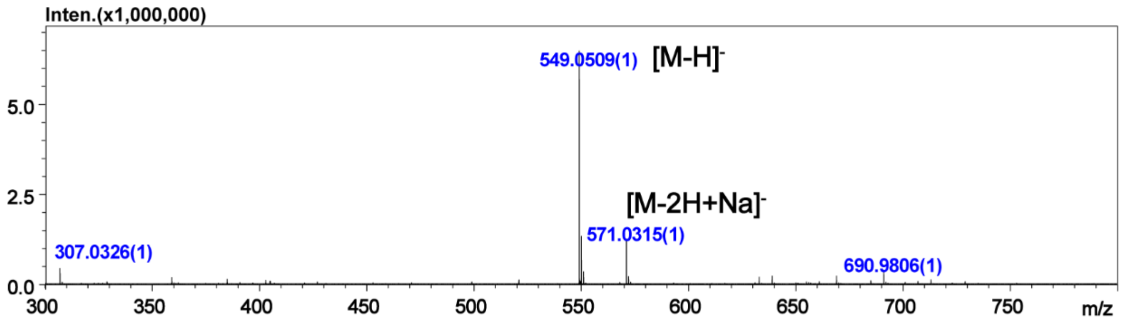

Supplementary Figure S8. HR-ITTOF-ESI/MS analysis of dUDP-D-Glc (M_w 550.0601) synthesized by Ss-RmlA.

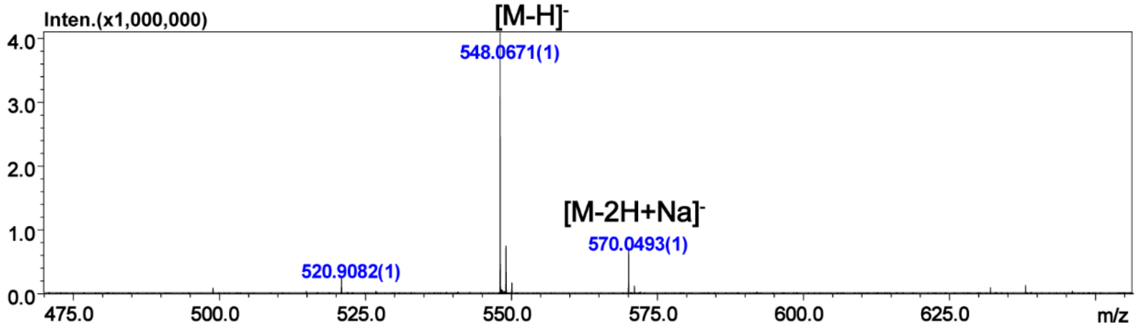

Supplementary Figure S9. HR-ITTOF-ESI/MS analysis of dUDP-D-GlcNH$_2$ (M_w 549.0761) synthesized by Ss-RmlA.

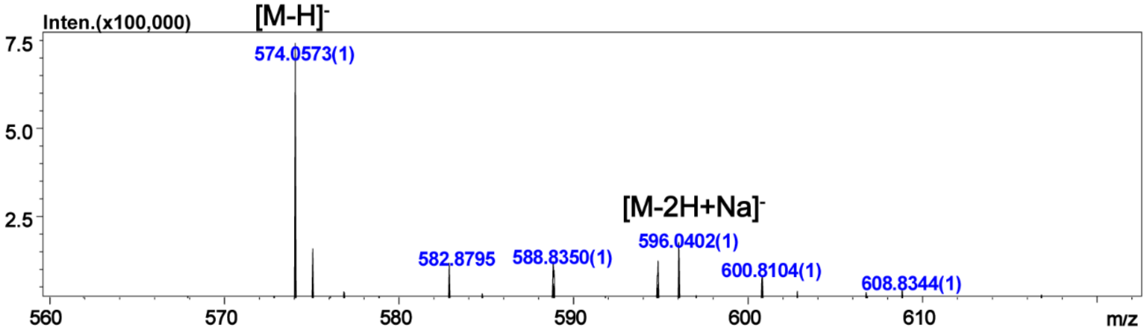

Supplementary Figure S10. HR-ITTOF-ESI/MS analysis of dUDP-D-GlcN$_3$ (M_w 575.0666) synthesized by Ss-RmlA.

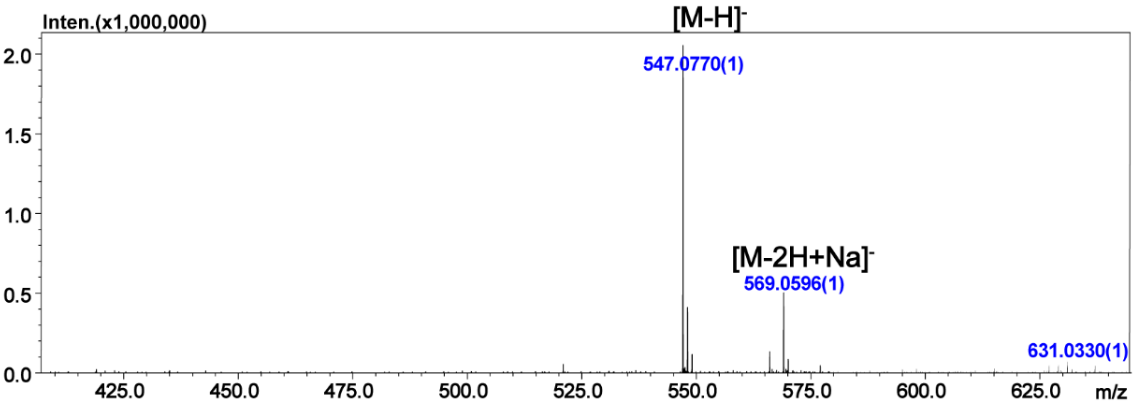

Supplementary Figure S11. HR-ITTOF-ESI/MS analysis of dTDP-L-Rha (M_w 548.0808) synthesized by Ss-RmlABCD in the one-pot reaction.

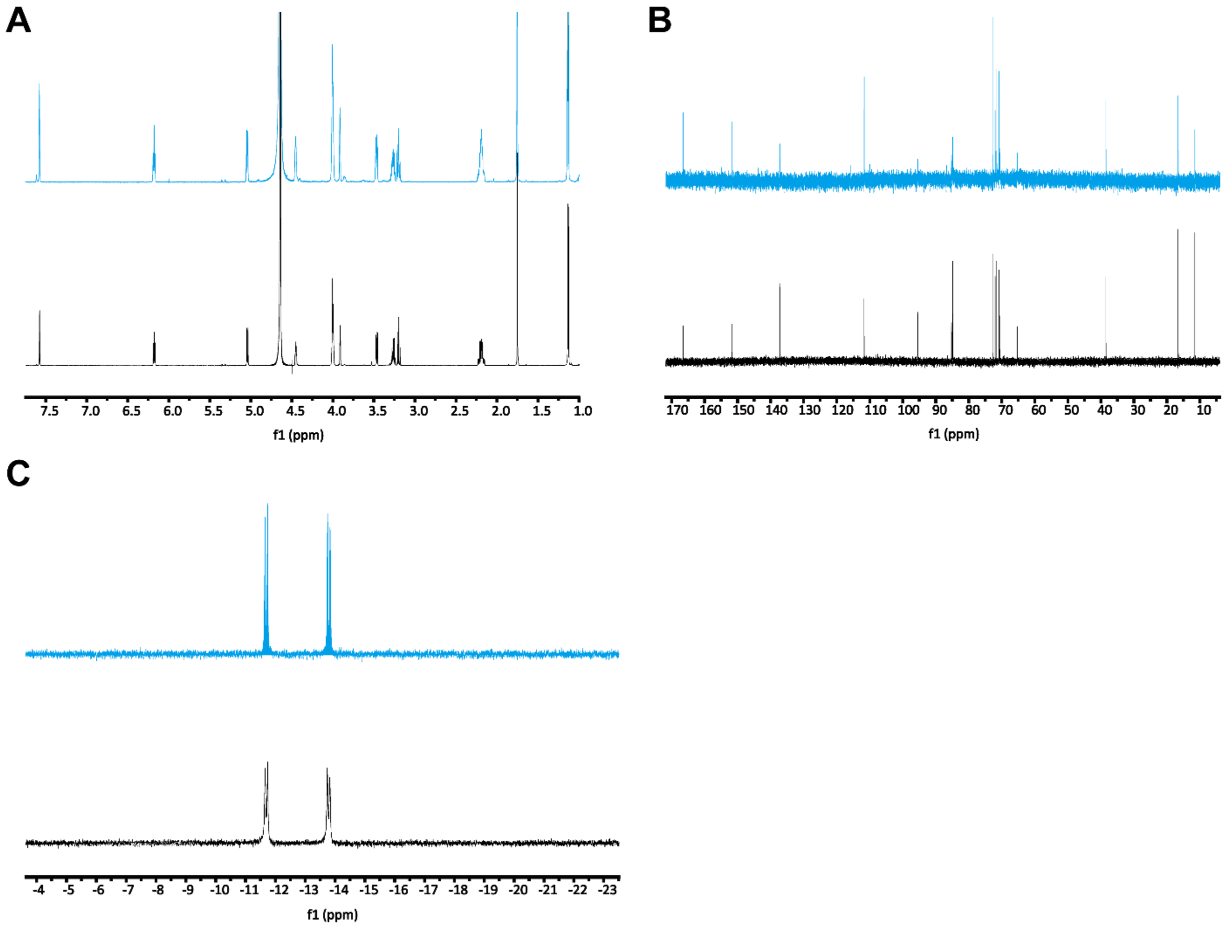

Supplementary Figure S12. Comparison of ^{1}H, ^{13}C and ^{31}P NMR spectra of published dTDP-L-Rha (blue) and dTDP-L-rhamnose synthesized by Ss-RmlABCD in this study (black). **(A)** ^{1}H NMR spectra; **(B)** ^{13}C NMR spectra and **(C)** ^{31}P NMR spectra. The NMR data were collected on an Agilent DD2 600-MHz instrument at room temperature in D_2O at 600 MHz for ^{1}H, 125 MHz for ^{13}C and 242 MHz for ^{31}P. Chemical shifts are given in parts per million (ppm) downfield from the internal TMS of D_2O.

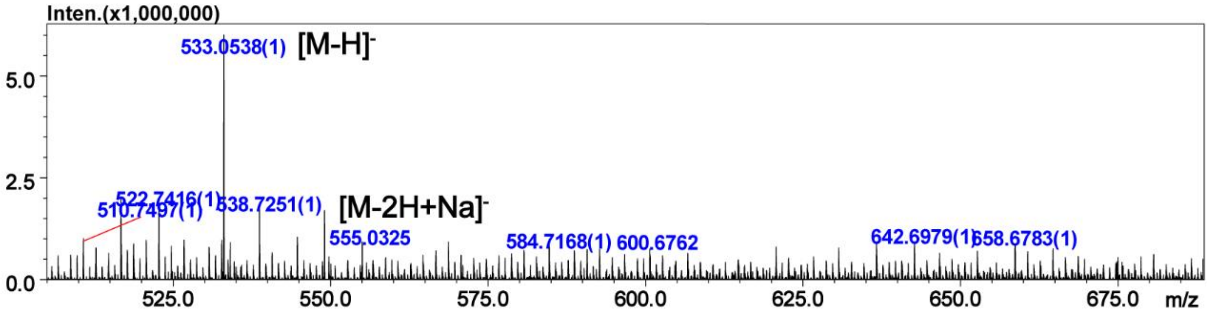

Supplementary Figure S13. HR-ITTOF-ESI/MS analysis of dUDP-L-Rha (M_w 534.0652) synthesized by Ss-RmlABCD in the one-pot reaction.

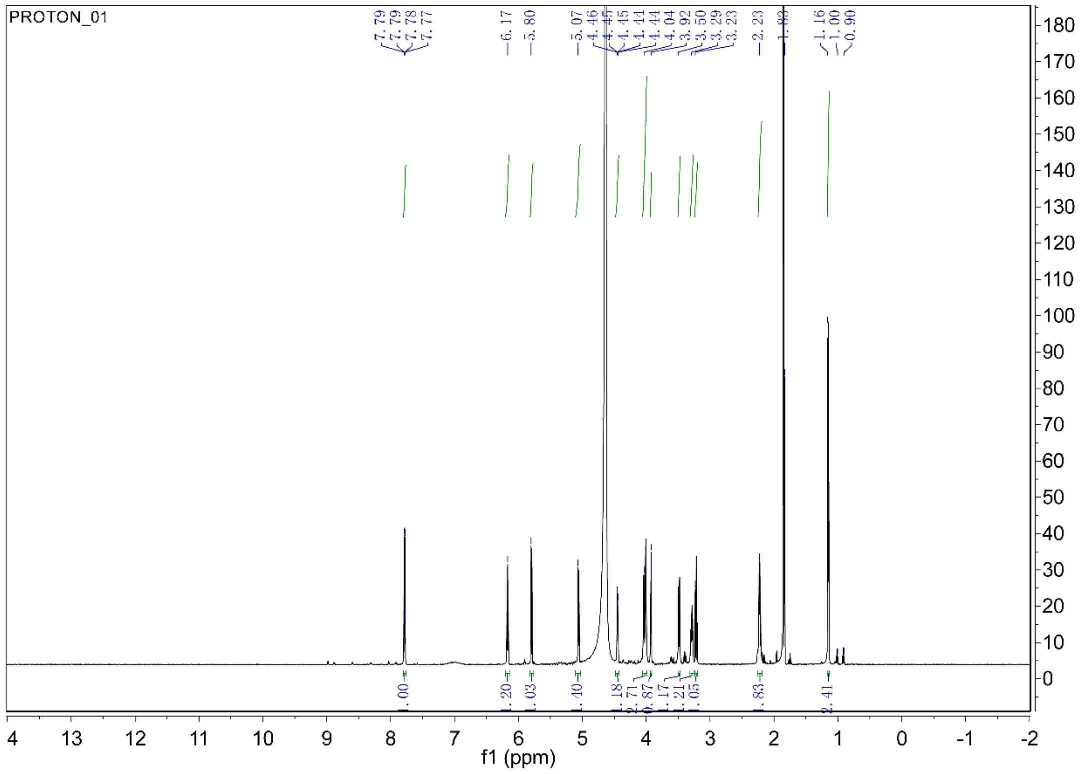

Supplementary Figure S14. ^{1}H NMR analysis of purified dUDP-L-Rha**.**

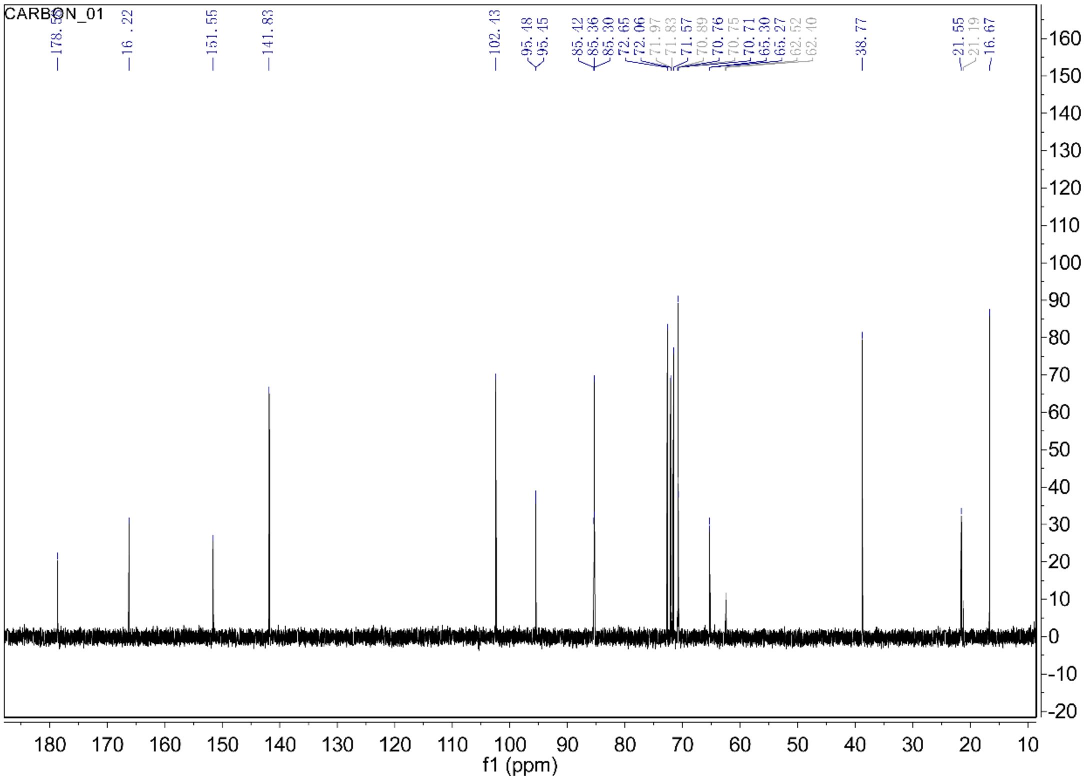

Supplementary Figure S15. ^{13}C NMR analysis of purified dUDP-L-Rha**.**

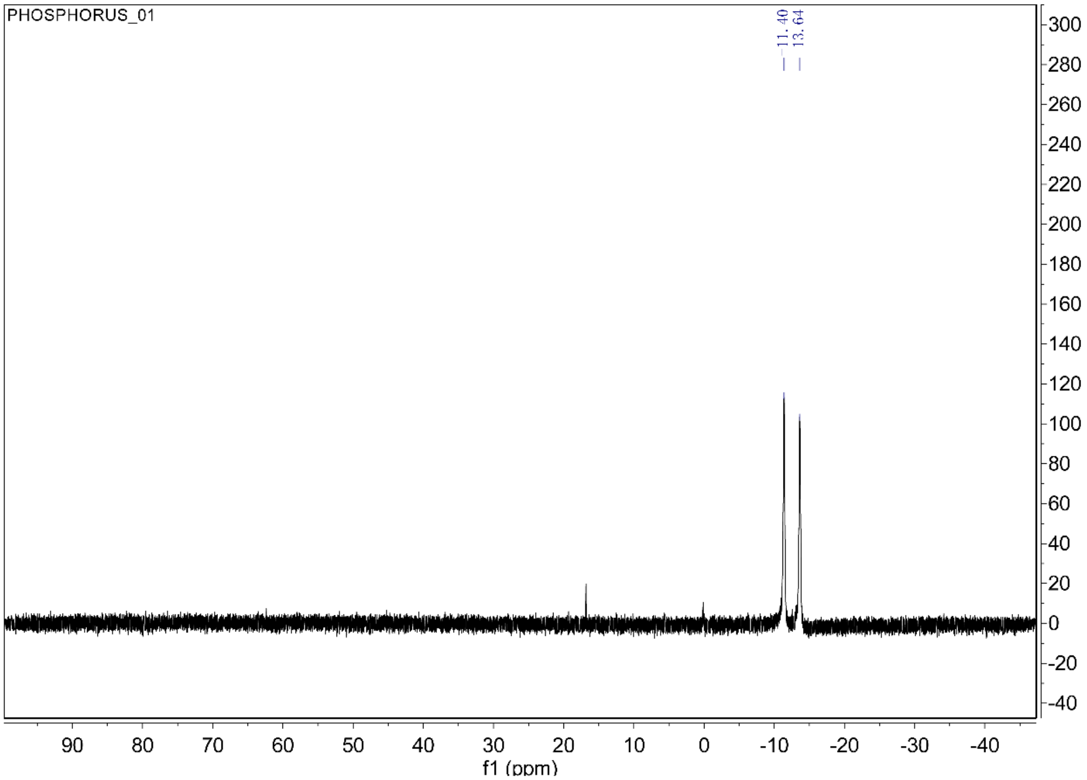

Supplementary Figure S16. ^{31}P NMR analysis of purified dUDP-L-Rha**.**

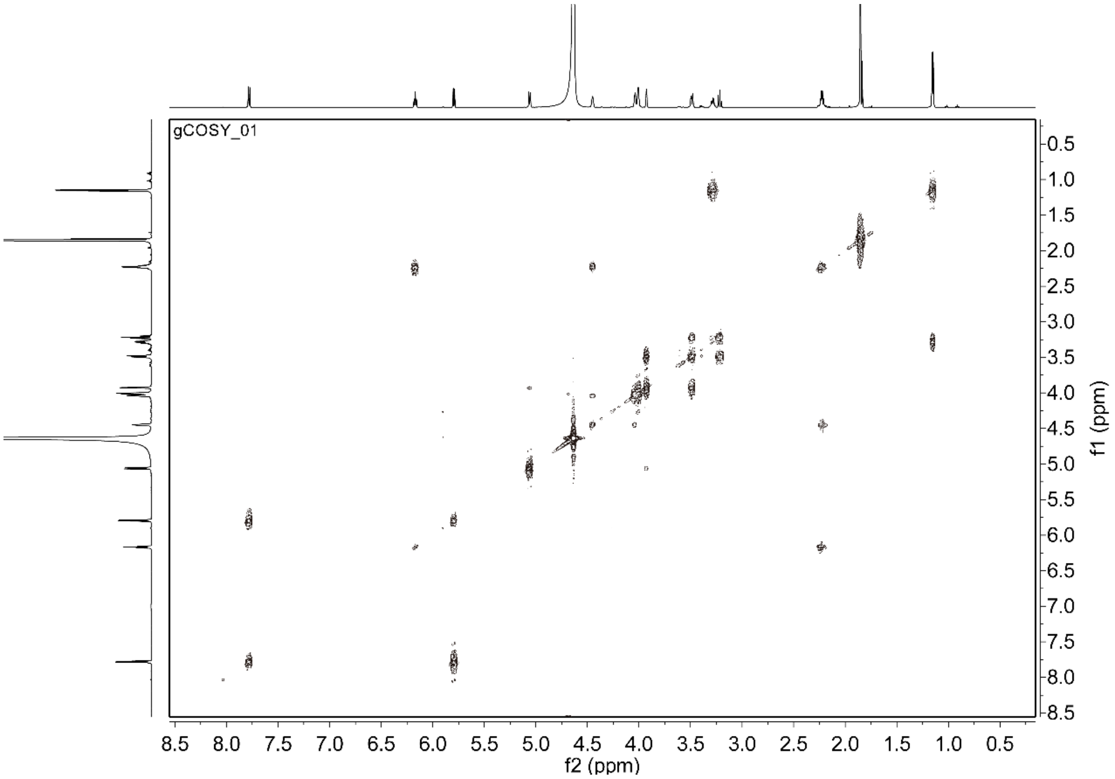

Supplementary Figure S17. ^{1}H-^{1}H COSY NMR analysis of purified dUDP-L-Rha**.**

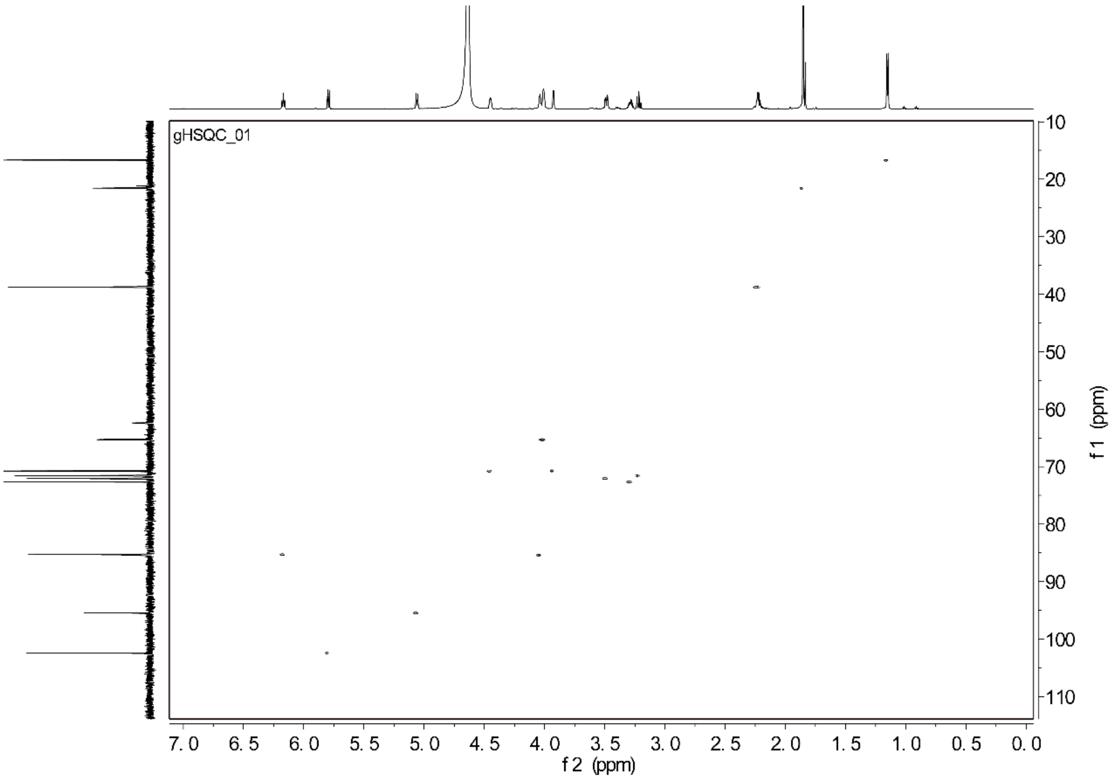

Supplementary Figure S18. ^{1}H-^{13}C HSQC NMR analysis of purified dUDP-L-Rha**.**

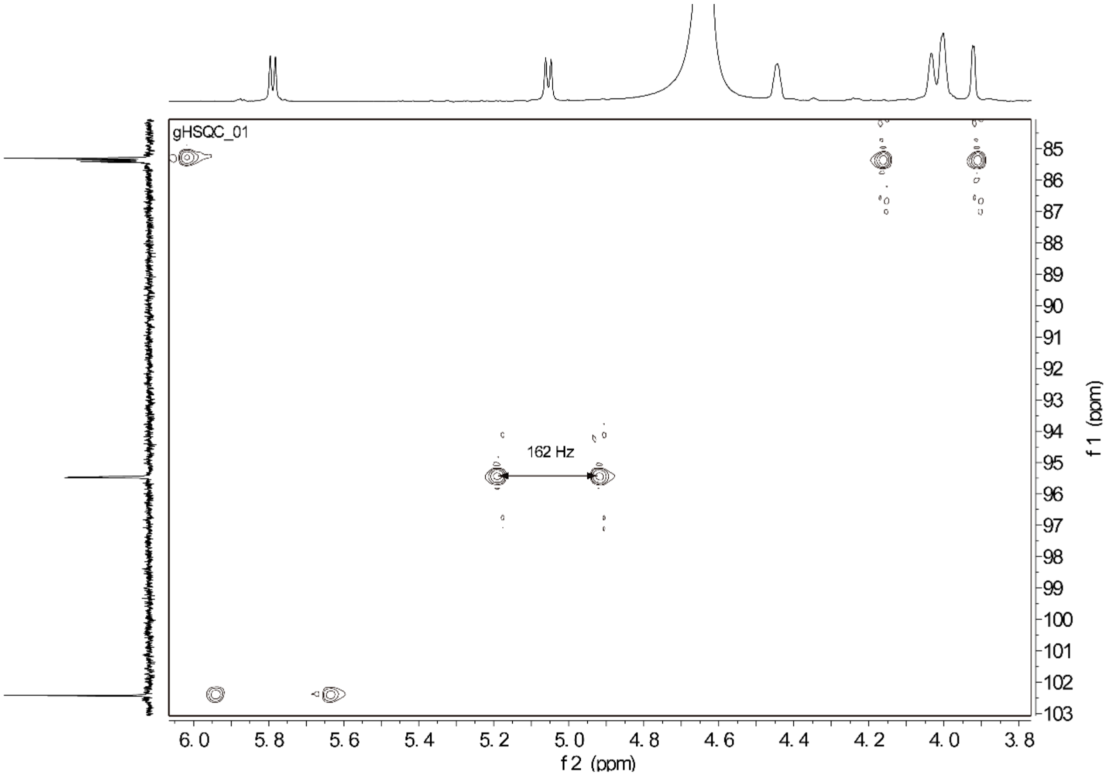

Supplementary Figure S19. ^{1}H-^{13}C HSQC without decoupling of purified dUDP-L-Rha**.**

Supplementary Table S1. Primers for amplification of the genes *Ss-rmlABCD* from *S. syringae* CGMCC 4.1716**.**

**Primers**	**Oligonucleotide sequences (5' - 3')**
Nde1-*S*s-*rml*A-F	ggaattcCATATGAAGGGCATCATCCTGGCCGGG
Xho1-*S*s-*rml*A-R	ccgCTCGAGGCGGACCTCGCGCGCGAC
Nde1- *S*s-*rml*B-F	gggaattcCATATGCGCGTGCTGGTAACGGG
Xho1- *S*s-*rml*B-R	ccgCTCGAGGCCGGTCAGCGCCGCG
Nde1-*S*s-*rml*C-F	ggaattcCATATGCAGGTTCGCCAACTCGC
Xho1-*S*s-*rml*C-R	ccgCTCGAGGCGGCCCCCCATCAAC
Nde1-*S*s-*rml*D-F	ggaattcCATATGGCGCTCGCGCTCCTGG
Xho1-*S*s-*rml*D-R	ccgCTCGAGGGGCACCGCCCGTGCC

The restriction sites were underlined and the flanking regions of primers were shown in lower case.
